# Development and testing of a novel compact system for municipal wastewater treatment and irrigation using advanced technologies

**DOI:** 10.1038/s41598-025-29122-y

**Published:** 2025-12-08

**Authors:** Abdel-hameed M. El-Aassar, Heba Isawi, Mohamed E. A. Ali, Hosam A. Shawky, Mohamed T. Mito, Selda Oterkus, Erkan Oterkus

**Affiliations:** 1 Water Treatment and Desalination Unit, Hydrogeochemistry Department, Water Resources and Desert Soils Division, Desert Research Centre, P.O.B. 11753, Cairo, Egypt; 2 Egypt Desalination Research Centre of Excellence, Desert Research Centre, Cairo, 11753 Egypt; 3 Mechanical Engineering Department, College of Engineering and Technology, Arab Academy for Science, Technology and Maritime Transport, Abu-Qir, Alexandria, Egypt; 4 Naval Architecture, Ocean and Marine Engineering, University of Strathclyde, Glasgow, UK

**Keywords:** Wastewater treatment, Crop irrigation, Membrane bioreactor, Moving bed biofilm reactor, Compact unit, Process chemistry, Chemical engineering

## Abstract

Growing water scarcity and increasing food demand are driving interest in municipal Wastewater Treatment (WWT) for crop irrigation. However, centralised WWT systems require substantial capital and infrastructure investment, which poses challenges for small agricultural communities. Decentralised compact systems are simpler to operate, more cost-effective, and better suited to rural areas. This study presents a novel, compact municipal WWT system specifically designed for crop irrigation. The system utilises four WWT technologies, integrating an ultrafiltration Membrane Bioreactor with mechanical sieving, extended aeration, and a Moving Bed Biofilm Reactor. A detailed sampling and testing methodology was implemented to experimentally evaluate system performance. The results showed significant reductions in major ions, with Total Dissolved Solids decreasing from 2348.8 to 1157.2 mg/L (50.7% reduction), and removal rates ranging from 25 to 98.9% for heavy metal ions from the municipal wastewater. The sanitary biological quality of the wastewater also improved substantially, with coliform and total microbial counts reduced by 81% and 18%, respectively. Overall, the product water met the Egyptian irrigation standards for edible crops, indicating the potential of this compact system as a practical solution for municipal WWT in rural areas.

**Supplementary Information:**

The online version contains supplementary material available at 10.1038/s41598-025-29122-y.

## Introduction

Water security is considered a major challenge due to the rapid increase in water consumption across the municipal, industrial and agricultural sectors^1,2^. This has increased interest in wastewater reuse to mitigate the growing water security challenges^3,4^. In early civilisations, raw sewage was sent to natural water bodies due to their ability to dilute and dissipate sewage. This was a practical method to dispose of small amounts of wastewater from small populations. However, large amounts of organic and inorganic waste found in wastewater can cause a significant reduction in oxygen levels and a high rate of aquatic life mortality^5,6^. Wastewater rich in untreated nutrients such as nitrogen and phosphorus can also promote rapid aquatic plant growth and eutrophication, in addition to pathogens that cause water-borne illnesses^5^. In the present era, with the increase in water demand and industrial development, it is essential to use suitable treatment facilities to decrease the effect of wastewater on the environment and ensure sufficient water for agriculture and food production, as demonstrated across several studies^7–16^.

Wastewater Treatment (WWT) has expanded in recent decades through the use of centralised sewage treatment plants to manage municipal wastewater and reduce the dependence and depletion of natural water sources^17,18^. However, municipal wastewater collection and treatment networks remain inadequate in many low- and middle-income countries, particularly in pre-urban and rural areas. The majority of wastewater from homes and businesses is released either untreated or only after minimal treatment^19,20^. Ideally, large amounts of wastewater from rural areas should be handled by centralised WWT facilities, which employ cutting-edge collection and treatment techniques^21^. However, installing such systems in small towns or rural areas may burden local governments financially, as they require large capital investments in sewer infrastructure, i.e., pumping stations, pipelines, deep excavations, and the numerous access hatches required for centralised production^22,23^. Accordingly, decentralised compact WWT systems are thought to be the best option for treating wastewater in remote villages or low-density agricultural communities^24^. Compared to centralised systems, decentralised systems are simpler to operate, less expensive, and better suited for low-density populations^13,25,26^. However, the main challenge is to design compact WWT systems that produce water that meets regulatory requirements and the right chemical properties for agricultural use^27^.

**Fig. 1:**
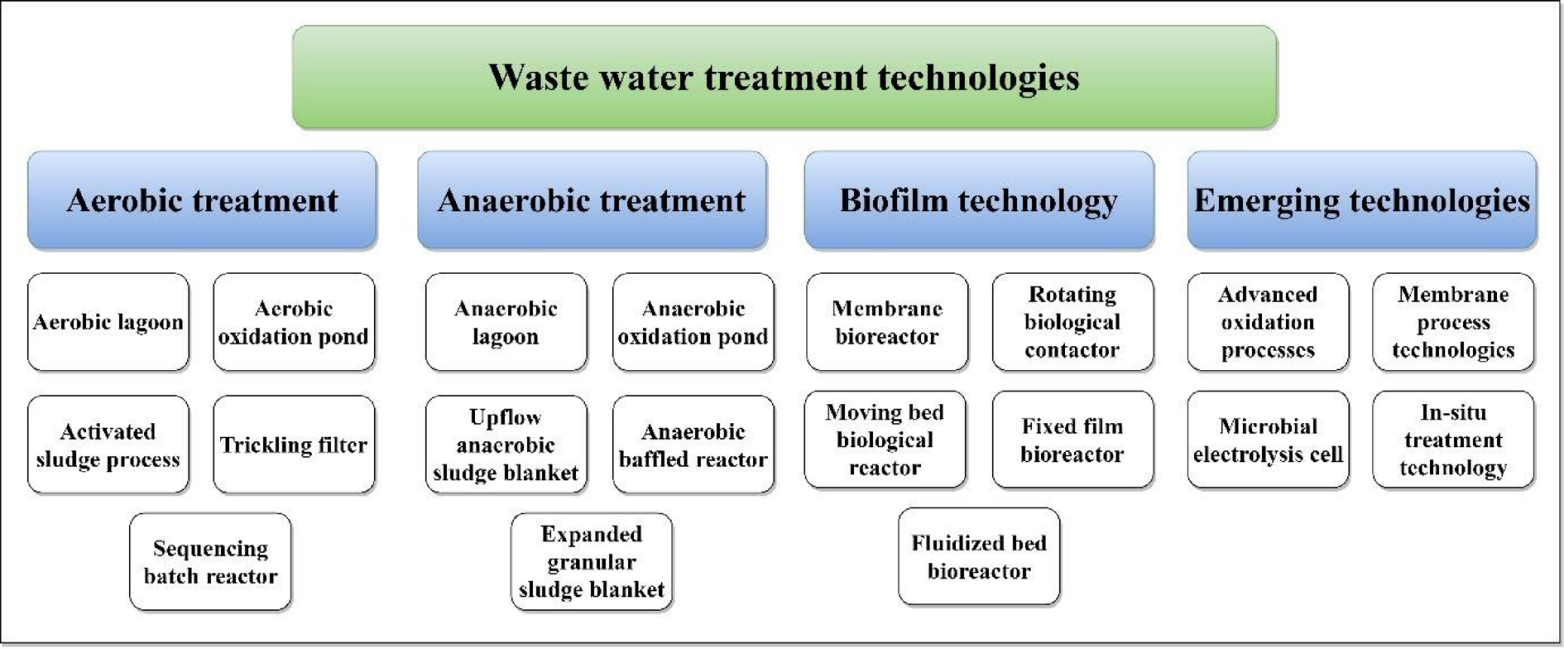
Wastewater treatment technologies (redrawn from Ref.^17^).

Various conventional and emerging WWT systems are currently used for treating industrial and municipal wastewater^17^. These systems can be categorised into aerobic treatment, anaerobic treatment, biofilm-based technologies, and emerging technologies, as illustrated in Fig. 1. Further details regarding their categorisation and operation are available in^17^. This study focuses on the application of Membrane Bioreactor (MBR) technology for the development of a compact WWT system capable of producing water suitable for agricultural reuse.

Over the past few decades, MBR has emerged as one of the most promising technologies for WWT and reuse^28^. MBR combines biological processes (aerobic and anaerobic), such as suspended fine growth bioreactors, with membrane filtration techniques such as microfiltration or ultrafiltration^29^. Unlike conventional biological treatments that rely on a clarifier for gravity settling, this technique separates the sludge created by biological treatments using microfiltration or ultrafiltration^30^. To put this technique into practice, Dorr-Oliver Inc. combined an activated sludge bioreactor with a cross-flow membrane filtration loop in the late 1960s. The procedure uses layers of polymeric flat sheet membranes with porosities varying from 0.003 to 0.01 µm^31,32^.

MBR offers several advantages over the conventional activated sludge process. It operates with a shorter Hydraulic Retention Time (HRT) but a longer Solid Retention Time (SRT), resulting in more effective sludge separation. Additionally, MBR produces higher-quality effluent with lower suspended solids, turbidity, and Biochemical Oxygen Demand (BOD), enabling a more compact system design and making it particularly suitable for water reuse^33^.

Several advancements have been made to MBR in terms of membrane configurations, materials, and operating parameters to expand its use in commercial applications^28^. This led to its use in many medium- to large-scale plants since the 1990s due to advancements in submerged configuration and efficient membrane materials. Globally, more than 5000 WWT facilities use MBR technology. However, its main drawback compared to conventional technologies is the higher capital and operating costs, along with the need for regular cleaning required to prevent membrane clogging^17^. This shows that further improvements are required to enhance its cost-effectiveness and sustainability^34^.

This study aims to develop and test a novel, compact, and decentralised WWT system based on MBR technology, specifically designed for agricultural communities. The system is capable of treating municipal wastewater with oil, grease, various organic compounds, and biological pollutants to produce water suitable for irrigation and sludge for potential biofuel production^27^. The study emphasises experimental investigation to generate real and reliable data rather than relying on predictive models to forecast plant performance^35^.

The compact WWT system combines four WWT technologies: 1) mechanical sieving, 2) extended aeration, 3) Moving Bed Biofilm Reactor (MBBR) biological treatment, and 4) MBR, which includes ultrafiltration membranes. Ultrafiltration delivers macromolecular separation for particles in the 20 to 1,000 Angstrom range (up to 0.1 microns). Large organic molecules, proteins, colloids, and microbiological pollutants are among the substances rejected by the membranes^36^. The objectives of the study are as follows:

1. Develop a novel compact WWT system design based on ultrafiltration MBR technology.
2. Fabricate, assemble, and commission the system with an emphasis on affordability and accessibility for local communities.
3. Collect and analyse municipal wastewater samples from an appropriate test site.
4. Develop and implement an experimental testing procedure for the compact unit to ensure that the treated water meets agricultural reuse standards.

This paper is structured as follows: Section “System design and implementation” describes the system design and implementation, including the concept and design, materials and fabrication, the wastewater chemical analysis performed at each treatment stage, and experimental methodology. Section “Results and discussion” outlines the results and includes the analysis and discussion for the experimental investigation. Section “Conclusions” provides the main conclusions.

## System design and implementation

### Concept and design

**Fig. 2:**
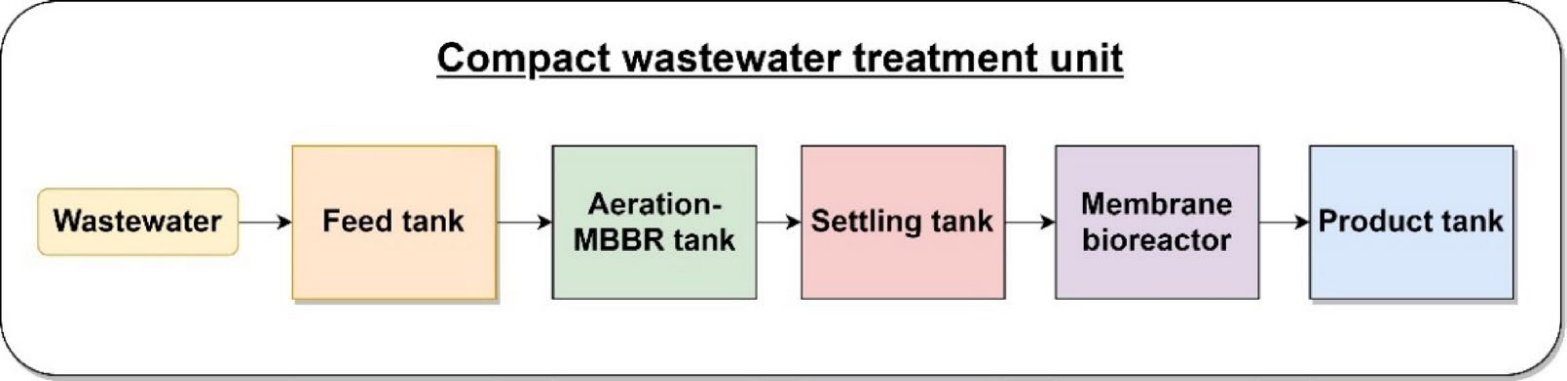
A block diagram describing the flow through the compact unit.

**Fig. 3:**
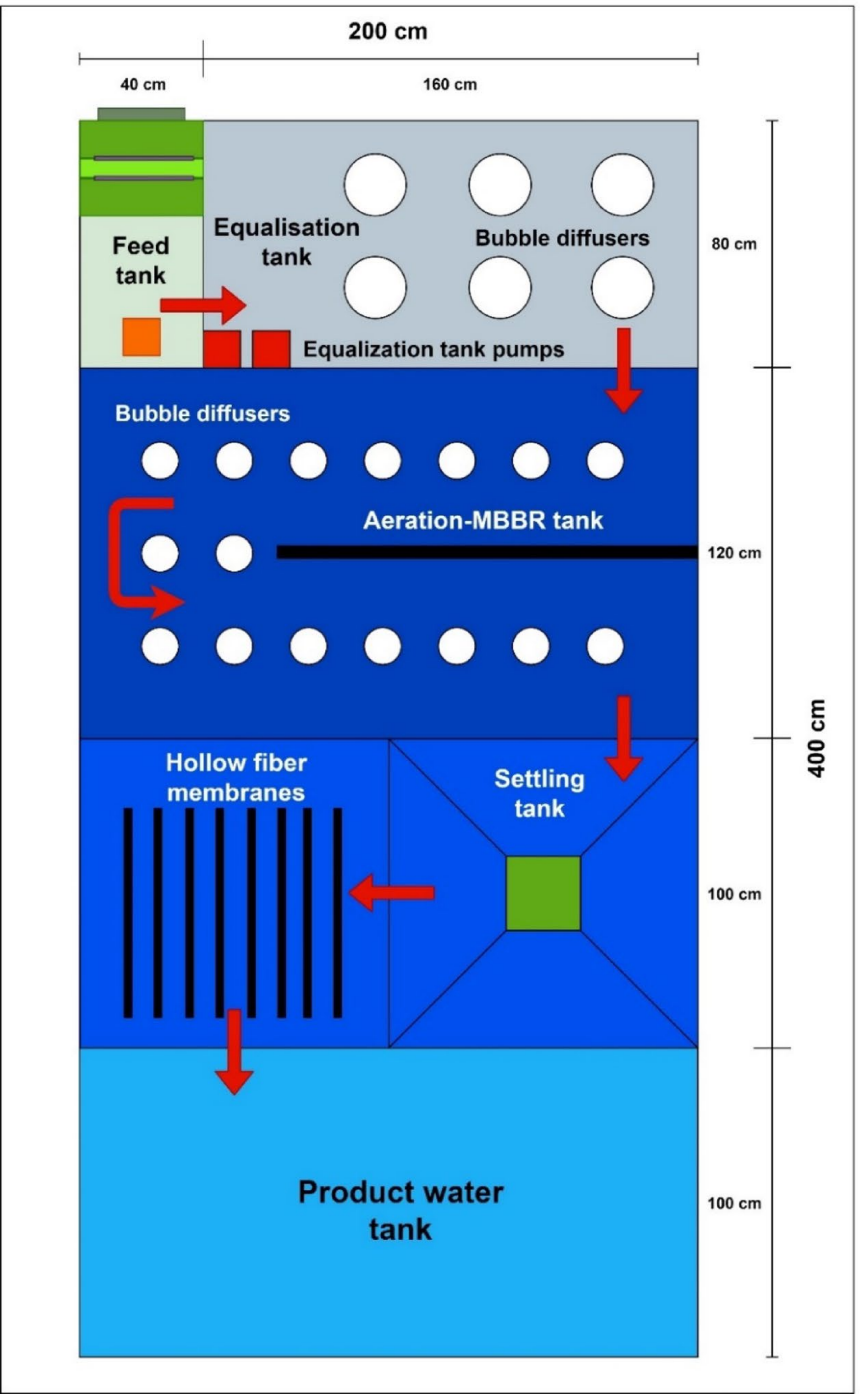
The schematic diagram and dimensions of the compact unit.

The compact WWT system combines four treatment techniques: mechanical sieving, extended aeration, MBBR biological treatment, and MBR ultrafiltration. Overall, the unit includes six tanks: a primary sedimentation feed tank, an equalisation tank, a combined extended aeration and MBBR treatment tank, a settling tank, an ultrafiltration membrane tank, and a product water tank. The retention time is four hours from the inlet of the wastewater to the outlet of the product water tank. A schematic of the wastewater flow through the compact unit is shown in Fig. 2, while the complete system design is illustrated in Fig. 3, with red arrows indicating the flow direction. The function of each tank, in the order of the process flow, is as follows:

1. The feed tank functions as the primary sedimentation tank. It is equipped with a 3 mm mechanical sieve to remove macromolecules and large colloids, as well as a funnel for separating oil and grease from the wastewater. Additionally, it serves as an overflow outlet for the compact unit.
2. The equalisation tank is used to equalise the organic load of feed wastewater. This is carried out using two air diffusers at the bottom and a fixed mixer at the top of the tank.
3. The combined extended aeration and MBBR tank includes six fine-bubble diffusers placed at the bottom, intended to offer prolonged aeration of the wastewater at a rate of 25 m^3^/hr of air at 0.2 bar gauge for each 1 m^3^/hr of wastewater fed to the compact unit. The diffusers’ bubbles keep the wastewater suspended and provide sufficient aeration, preventing excessive sludge development. Cylindrical High-Density Polyethylene (HDPE) bio-carriers with cross fins and a high specific surface area (~ 500–800 m^2^/m^3^) are added as growth media to allow effective microbial attachment and biofilm formation for enhanced biological degradation of organic matter. Because these carriers provide a large surface area for microbial colonisation, the bacteria can produce enzymes more efficiently and break down organic matter more rapidly. The MBBR treatment principle is followed by this mix of attached growth (biofilm on medium) and suspended growth (aeration), which enhances overall treatment efficiency.
4. The settling tank consists of a cone-shaped bottom and is used for settling any macromolecules to prevent clogging of the hollow fibre membrane element.
5. The fifth tank is for MBR WWT using the ultrafiltration hollow fibre membrane elements made from Polyvinylidene Fluoride (PVDF). The membranes measure 48 cm × 112.7 cm and were supplied by Shanghai Haozun Industry Co., Ltd. (China). They are specifically designed for municipal WWT, providing an effective barrier against suspended solids, bacteria, and pathogens, ensuring safe reuse for irrigation. Two membrane elements were used, each operating at a flux of 0.5 m^3^/hr.
6. The last tank is the product water tank, which stores the treated wastewater.

As shown in Fig. 3, the compact unit is equipped with multiple pumps, including two blowers for aeration, an outlet pump, and a submersible pump. It also features an integrated housing for the feed pump with a variable-speed drive, the main electrical panel, and a monitoring panel containing temperature, pressure, and flow meters. The unit is powered by a generator.

**Fig. 4:**
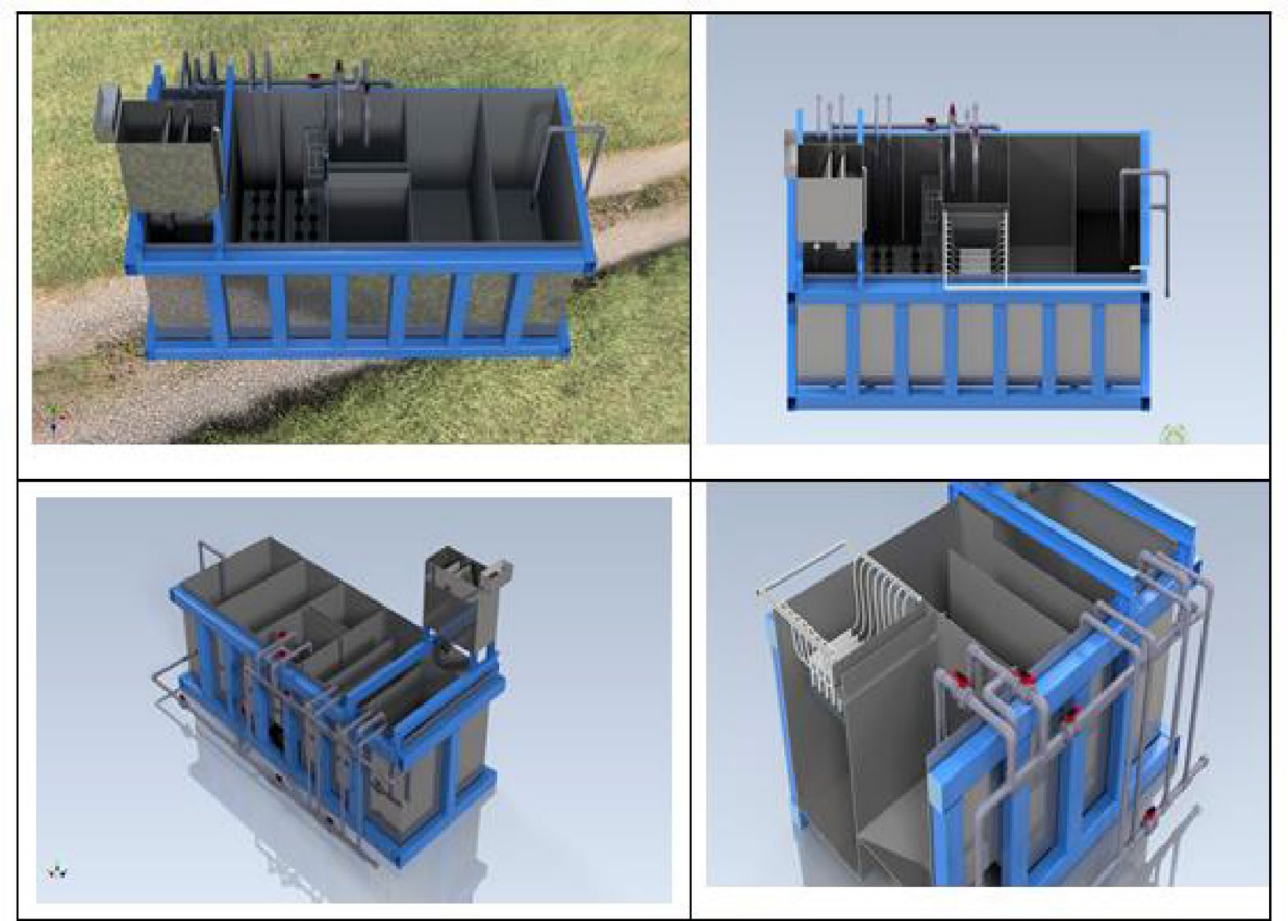
A 3D CAD of the compact wastewater treatment unit.

As shown in Fig. 4, the compact unit measures 2 m × 4 m × 1.8 m (width × length × height) and weighs around 3 tonnes when empty and 17 tonnes when filled with wastewater. The compact unit sizing and component selection target a production capacity of 20 m^3^/day of treated wastewater. It is also fitted with a rising suction system to extract solids and sludge from the settling tank in semi-liquid form, allowing for their potential use for biofuel generation^27^. Moreover, the compact unit operates on a cycle of two hours of treated water production followed by fifteen minutes of backwashing of the ultrafiltration membranes to minimise fouling.

In traditional WWT plants, filtration is used to remove particulates after secondary sedimentation. However, the MBR tank with ultrafiltration membranes replaces this stage in the current design, resulting in a high-quality effluent that can be reused for irrigation. Furthermore, despite the small system’s lack of a separate disinfection step, the MBR ultrafiltration membranes successfully lowered bacteria counts to satisfy Grade A irrigation requirements. An optional last disinfection step (such as UV or chlorination) could be included for full-scale deployment to offer lingering defense against microbial regrowth throughout distribution and storage.

### Materials and fabrication

**Fig. 5:**
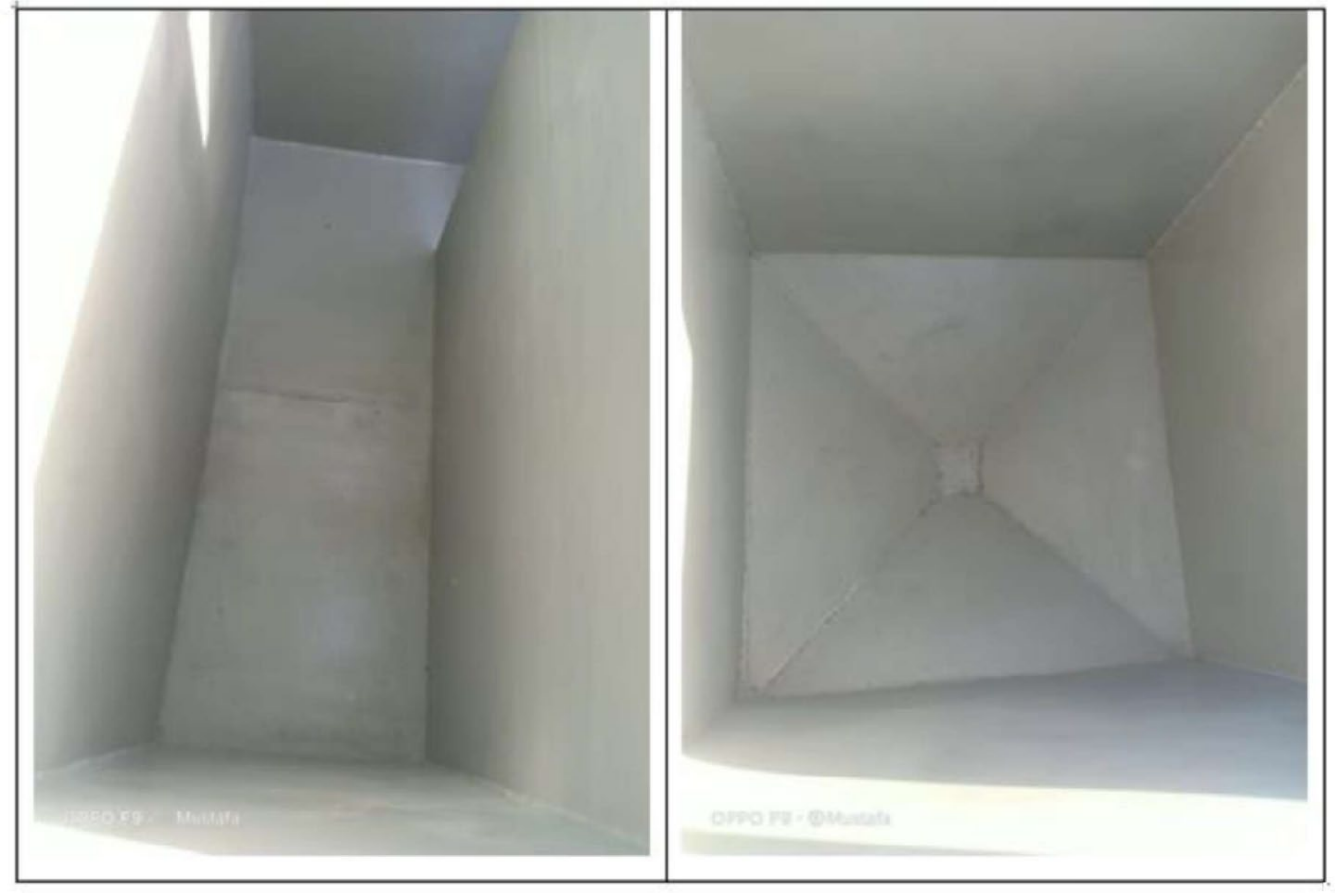
The internal coating of the compact unit with epoxy.

**Fig. 6:**
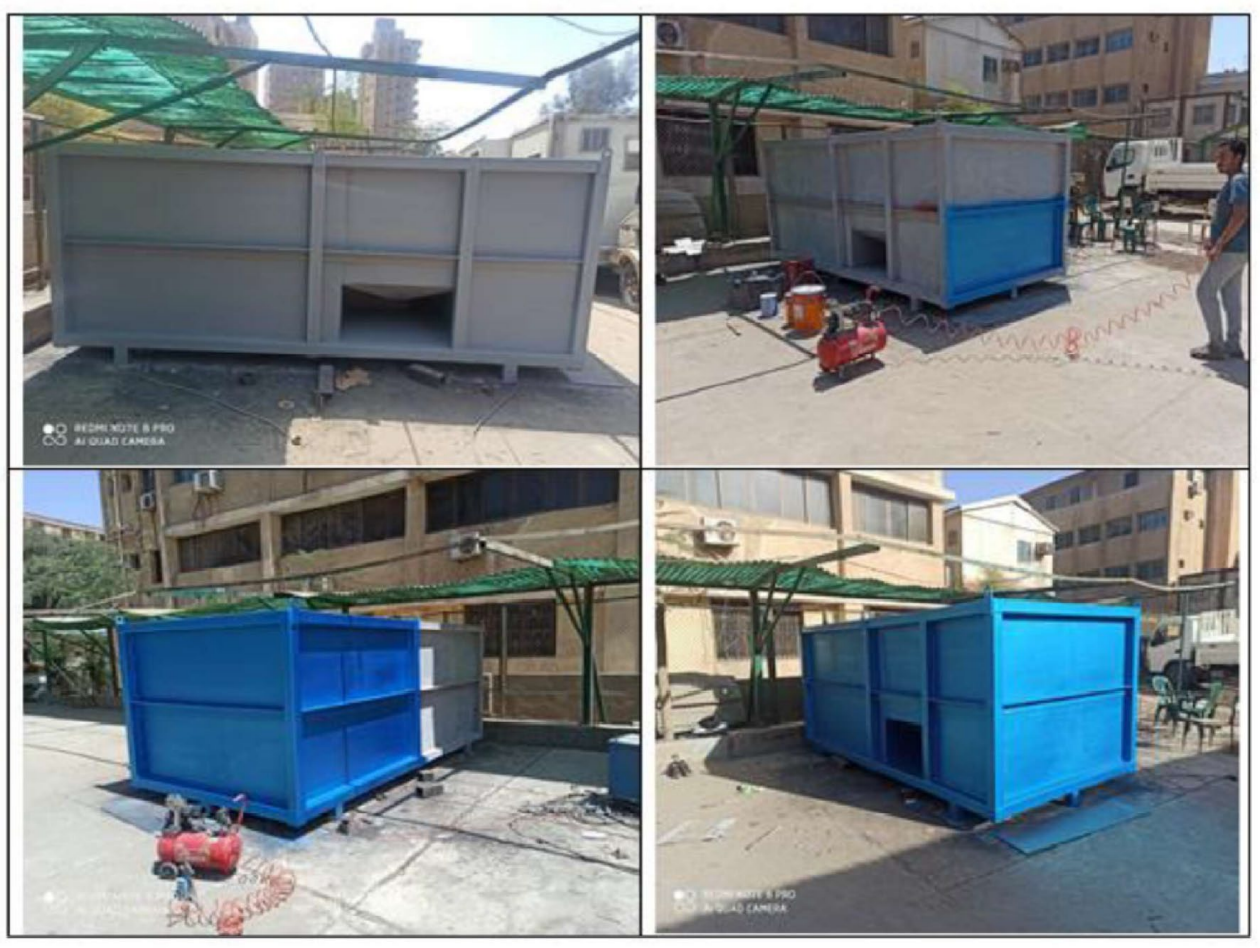
The external coating of the compact unit.

**Fig. 7:**
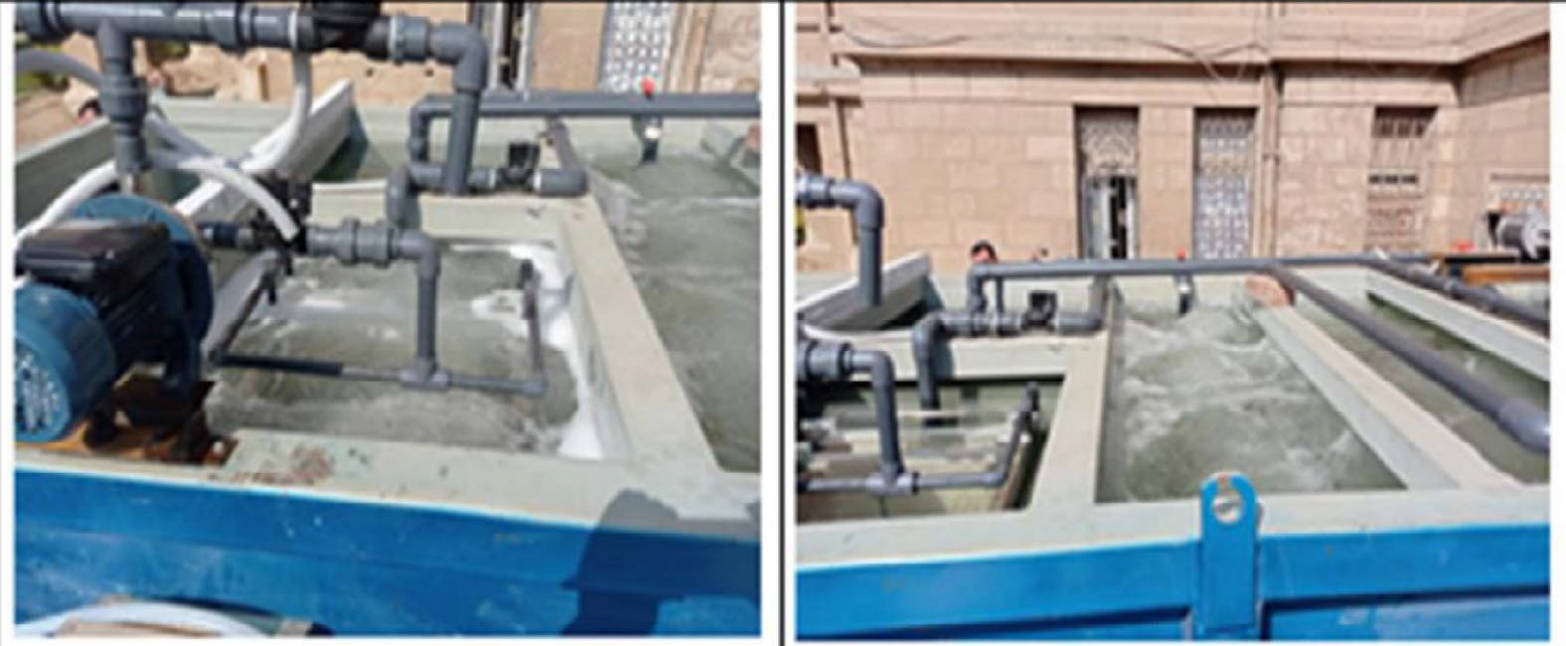
The installation and internal connection with blowers and hollow fibre elements.

**Fig. 8:**
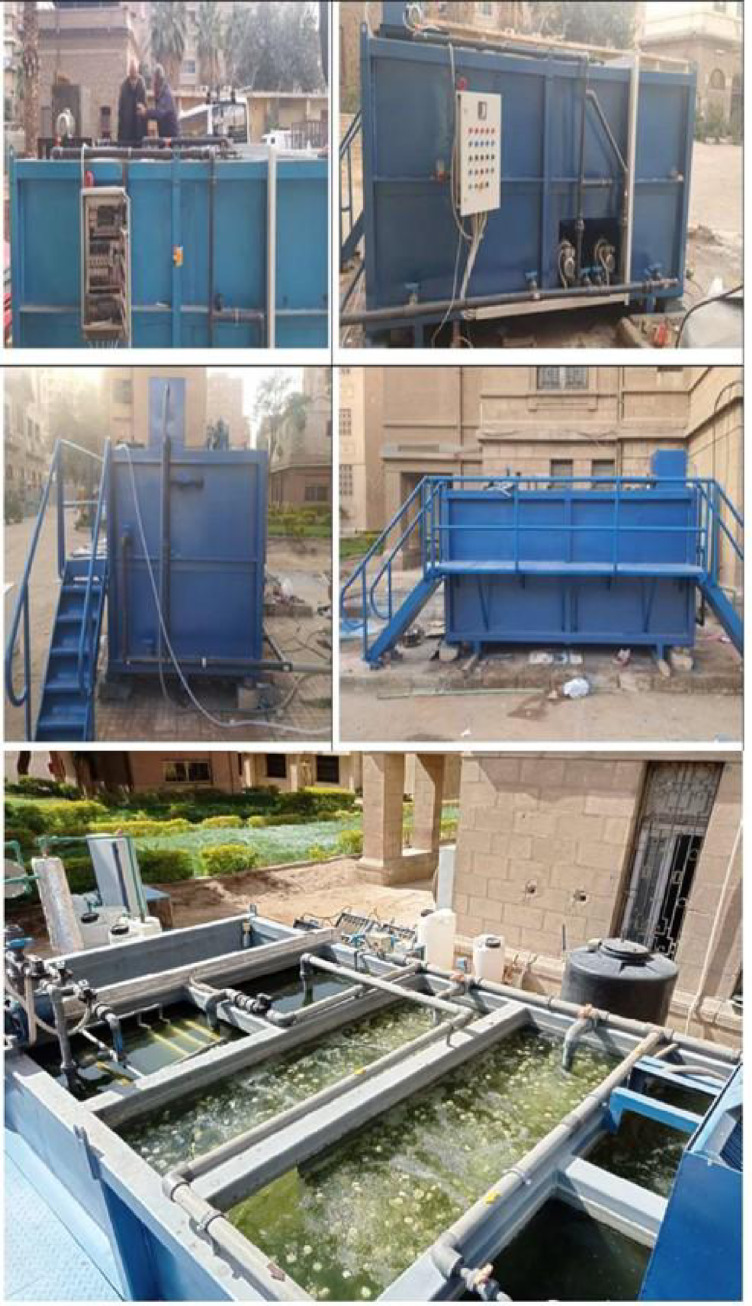
The complete assembly of the compact unit in operation during operation.

The fabrication procedure took several months and multiple discussions with companies in the field of WWT to implement the required design and achieve the desired performance, using local materials and equipment. The construction of the compact unit involved several steps. The structure is made of carbon steel that is coated internally with ceramic and epoxy to protect against corrosion and externally coated with metallic paint that is resistant to sun radiation and high temperature, as shown in Figs. 5 and 6, respectively. The internal connections and components, i.e., pipes and pumps, are then installed, as shown in Fig. 7. This was followed by installing the blowers and hollow fibre elements. After the setup of the compact unit was completed, as shown in Fig. 8, testing and experimentation were done using municipal wastewater samples.

### Wastewater chemical analysis

**Fig. 9:**
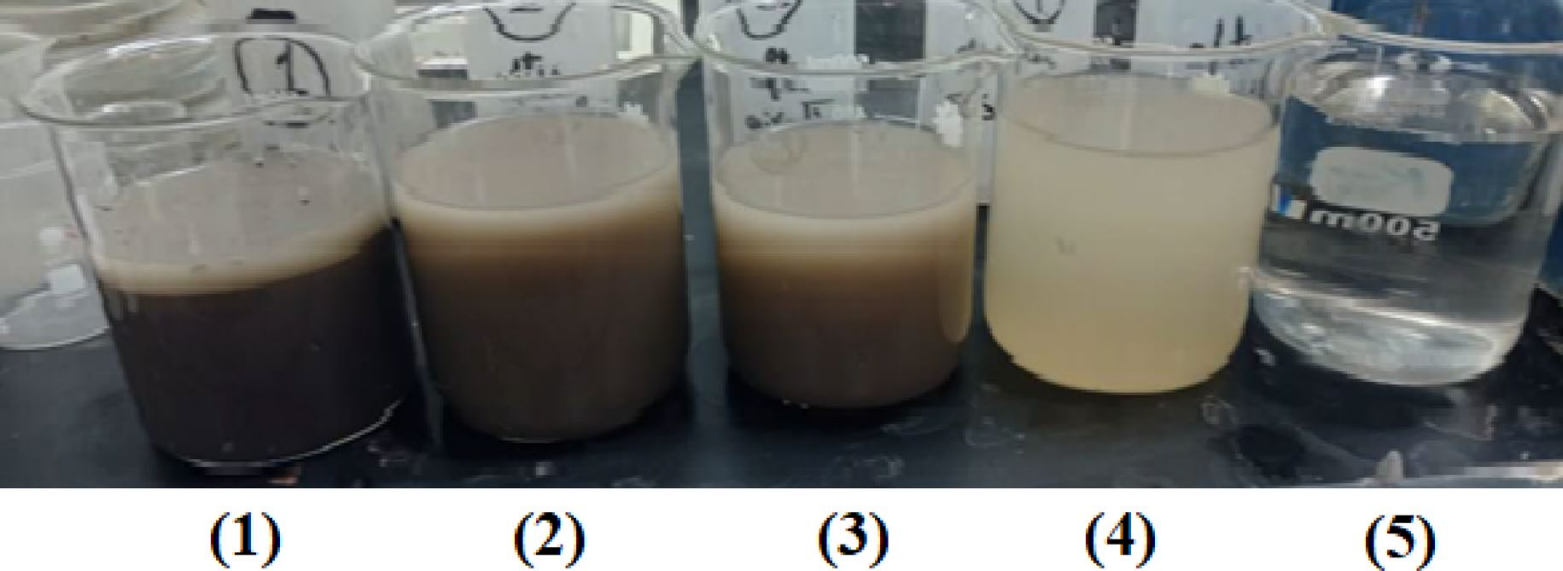
The collected water samples: (1) feed wastewater, (2) after equalisation tank, (3) after oxidation tank, (4) after final settling tank, and (5) after ultrafiltration process.

Municipal wastewater was collected from the Holding Company for Potable Water and Wastewater in Cairo and used as feed water for the compact unit. The wastewater samples were collected at each treatment stage for chemical analysis, as shown in Fig. 9. All required chemical analyses were performed in the laboratories of the Desert Research Centre (DRC).

Microbiological examination, heavy metals, biological pollutants, Electrical Conductivity (EC), TDS, Hydrogen Ion Concentration (pH), and the concentrations of cations (Na^+^, K^+^, Ca^2+^, Mg^2+^) and anions (Cl^−^, SO_4_^2−^, CO_3_^2−^, HCO_3_^−^) were among the analyses performed. The pH and EC were measured in the field using portable pH and EC meters (model Orion 150 A^+^). An aqueous solution’s EC is measured in microsiemens per centimetre (μS/cm at 25 °C), which represents its ability to conduct an electric current.

Water samples were preserved and analysed according to the accepted methodologies of Hem^37^, American Public Health Association (APHA)^38^, ASTM^39^, Fishman and Friedman^40^, and Rainwater and Thatcher^41^. The concentrations of the main ions Ca^2+^, Na^+^, Mg^2+^, K^+^, HCO_3_^−^, SO_4_^2−^, CO_3_^2−^, and Cl^−^ were included in the lab analysis. The minor and heavy metals analysed included P^3+^, B^3+^, Al^3+^, Fe^3+^, Mn^2+^, Co^2+^, Cu^2+^, Ni^2+^, Cr^3+^, Cd^2+^, Pb^2+^, Sr^2+^, V^2+^, and Zn^2+^, as well as Total Organic Carbon (TOC), Chemical Oxygen Demand (COD), and BOD. A Jenway PFP 7 flame photometer was used to estimate the Na^+^ and K^+^. AgNO_3_ titration was used to determine the Cl^−^. Titrimetric measurements of calcium (Ca^2+^) and magnesium (Mg^2+^) were made using the standard EDTA titration techniques. Volumetric methods were utilised to investigate CO_3_^2−^ and HCO_3_^−^. To determine SO_4_^2−^, the turbidimetric method was applied using a UV/Visible spectrophotometer (Thermo-Spectronic 300). The EC was multiplied by an aspect of 640 to calculate the TDS^42^. The obtained chemical data are presented as milligrams per litre (mg/L) or Parts Per Million (ppm). TDS (mg/L), the outcome of all chemical analyses, can be evaluated in the manner described below according to Hem’s 1991^37^ assessment:

1 $${\text{TDS}} = {\text{Ca}}^{2 + } + {\text{Mg}}^{2 + } + {\text{Na}}^{ + } + {\text{K}}^{ + } + {\text{CO}}_{3}^{2 - } + {1 \mathord{\left/ {\vphantom {1 2}} \right. \kern-0pt} 2}{\text{HCO}}_{3}^{ - } + {\text{SO}}_{4}^{2 - } + {\text{Cl}}^{ - }$$

An Inductively Coupled Argon Plasma (ICP) 6500 Duo spectrophotometer (Unicom, UK) was used to estimate the heavy metals. Titrimetric analysis was used to estimate the TOC. BOD is a biochemical technique that uses the 5-day BOD test to analyse organic material present in a given water sample and determine the amount of dissolved oxygen required by aerobic microorganisms in a body of water. The titrimetric approach, which calculates the amount of oxygen required to oxidise carbon-based materials in wastewater samples under a specific temperature, oxidising agent, and other conditions, was used to determine the COD. The UV/Visible Spectrophotometer, Unicam model UV4-200 (UK), with a wavelength of 543 nm, was used to measure nitrite (NO_2_^−^). Kjeldahl steam distillation was used to evaluate the levels of ammonia (NH_4_^+^) and nitrate (NO_3_^−^). Total Suspended Solids (TSS) were measured by passing a weighted volume of water through a pre-weighed strainer with a predetermined pore size. A Nephelometer, sometimes referred to as a turbidity meter, was used to estimate the turbidity in Nephelometric Turbidity Units (NTU).

In terms of assessment of the total coliforms and Escherichia coli in the samples, the Most Probable Number (MPN) method was used in accordance with the standard protocols recommended by the APHA^43^. Before being inoculated into lactose broth tubes with inverted Durham tubes, aliquots of each sample were serially diluted in sterile distilled water. For 24 to 48 h, the infected tubes were incubated at 37 ± 1 °C in order to identify total coliforms. The presumptive tests were recorded as positive for tubes showing acid and/or gas formation. For the confirmatory test, an aliquot was taken from each positive tube and placed in Brilliant Green Lactose Bile (BGLB) broth. The tubes were then incubated for another 24 h at 37 ± 1 °C. A loopful of positive BGLB tubes was streaked onto Eosin Methylene Blue (EMB) agar and incubated for 24 h at 44 ± 0.5 °C in order to detect E. coli. Colonies with a distinctive metallic green sheen were regarded as E. coli positive. Standard MPN statistical tables were used to determine the MPN of coliforms per 100 mL of sample, and the number of positive tubes at each dilution was noted^43^.

## Results and discussion

This section presents the analysis and discussion of the compact unit’s experimental results, reported as mean values from three replicate tests to ensure accuracy and repeatability. A comprehensive error analysis was conducted, with standard deviations calculated for all parameters. The raw data and the corresponding error analysis are provided in the Supplementary Information Files (Tables S.1 and S.2 for major ions and heavy metals, respectively).

### Analysis of major ions

**Fig. 10:**
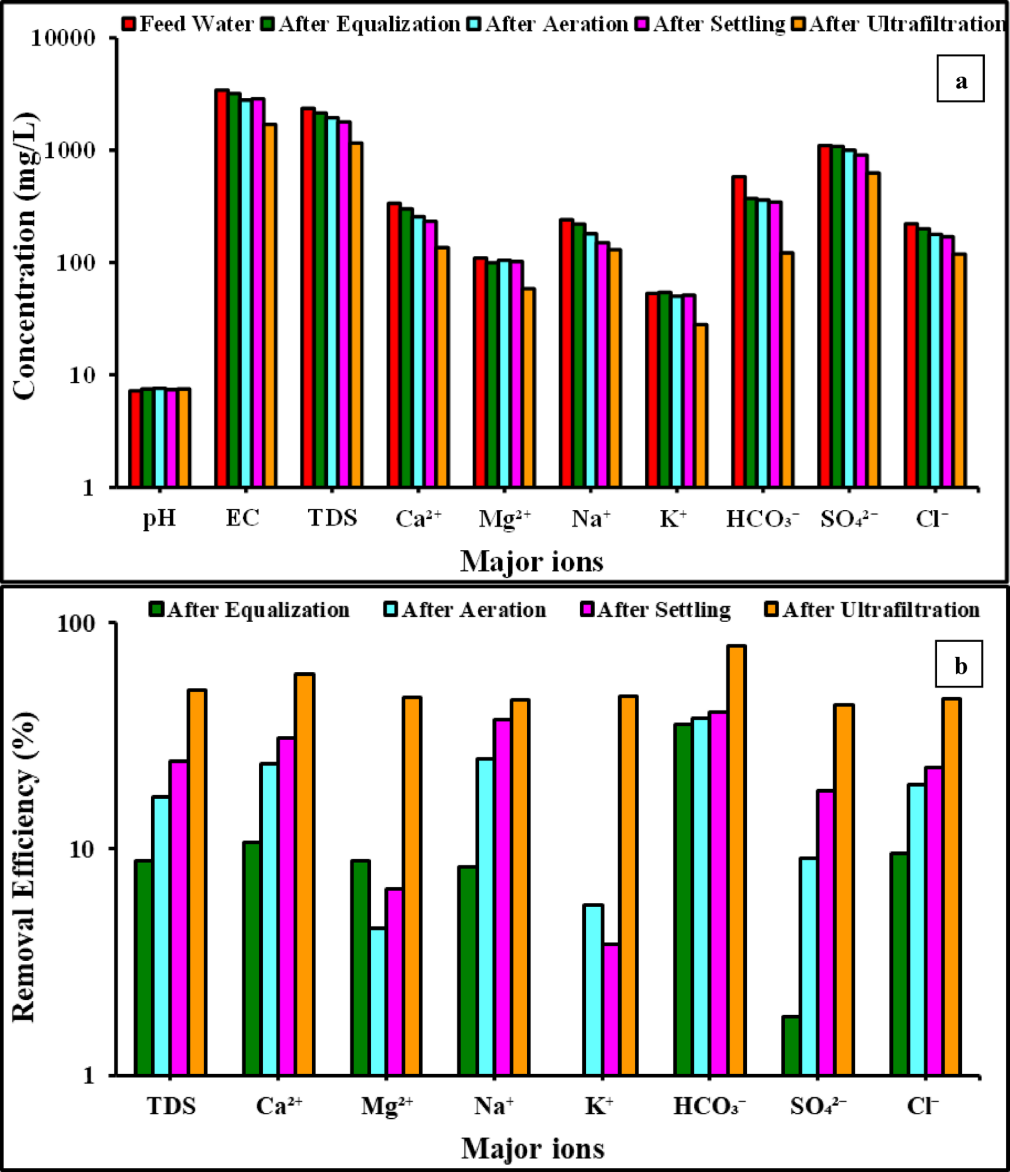
(**a**) Concentration (mg/L); (**b**) Removal efficiency (%) of pH, EC, and major ions (Ca^2+^, Mg^2+^, Na^+^ + K^+^, CO_3_^2−^ + HCO_3_^−^, SO_4_^2−^, Cl^−^) for the feed wastewater and after each stage (Equalisation, Aeration-Moving bed biofilm reactor, Settling, and Ultrafiltration) using municipal wastewater from the Holding Company for Potable Water and Wastewater in Cairo, Egypt. Y-axis shown in logarithmic scale.

The analysis carried out before the treatment process revealed that the concentrations of major cations and anions (Ca^2+^, Mg^2+^, Na^+^, K^+^, HCO_3_^−^, SO_4_^2−^, and Cl^−^) and the salinity of these chosen water samples were all higher than the WHO standard limits for irrigation, as shown in Fig. 10a. The initial wastewater sample had a TDS of 2348.8 mg/L; however, after going through four stages, it was reduced to 1157.2 mg/L (50.7% reduction), which is indicative of treated water suitable for various crop irrigation applications.

The chemistry of the water slightly changed after equalisation. Due to mixing and stabilisation effects, the pH slightly rose from 7.2 to 7.5. TDS and EC dropped from 2348.8 to 2139.2 mg/L and from 3420 to 3180 µS/cm, respectively, suggesting partial dilution or early sedimentation of suspended particles. The minor decrease seen in major cations (Ca^2+^, Mg^2+^, Na^+^, and K^+^) and anions (Cl^−^, SO_4_^2−^, and HCO_3_^−^) indicate that equalisation mainly encourages homogenization and partial settling of larger suspended particles rather than substantial ionic elimination. The water quality was further altered by aeration. As a result of oxygenation and CO_2_ removal, the pH increased slightly to 7.6. TDS and EC both dropped to 1948.7 mg/L and 2790 µS/cm, respectively. Significantly, the amounts of Ca^2+^ dropped to 256 mg/L, which is consistent with carbonate precipitation brought on by elevated pH and oxygenation. Likewise, sulfate was largely constant while bicarbonate and chloride decreased. These modifications demonstrate how well aeration works to remove carbonate hardness and encourage chemical precipitation. The pH slightly dropped to 7.4 after sedimentation, presumably as a result of the balance between carbonate buffering and CO_2_ absorption. The TDS continued to drop till reaching 1777.2 mg/L. Continued reductions in major ions, such as Ca^2+^ (232 mg/L), Mg^2+^ (102.06 mg/L), and Cl^−^ (169.8 mg/L), suggested that precipitated and suspended particles had been successfully removed. By allowing flocs created during aeration to settle gravitationally, this stage mainly improves clarity. The biggest gains were achieved in the ultrafiltration stage, in which the EC and TDS dropped to 1690 µS/cm and 1157.2 mg/L, respectively, representing a 50.7% reduction relative to the feed water. Ca^2+^ and Mg^2+^, two divalent cations, were significantly reduced to 136 mg/L and 82.36 mg/L, respectively, as shown in Fig. 10a. Cl^−^ and SO_4_^2−^ also decreased to 118.9 mg/L and 625 mg/L, respectively, indicating the membrane’s strong rejection efficiency for dissolved solids and salts. Effective buffering during filtration is suggested by the minor pH stability at 7.5. Overall, ultrafiltration was essential for enhancing the water quality, especially when it came to lowering salinity and ions.

**Fig. 11:**
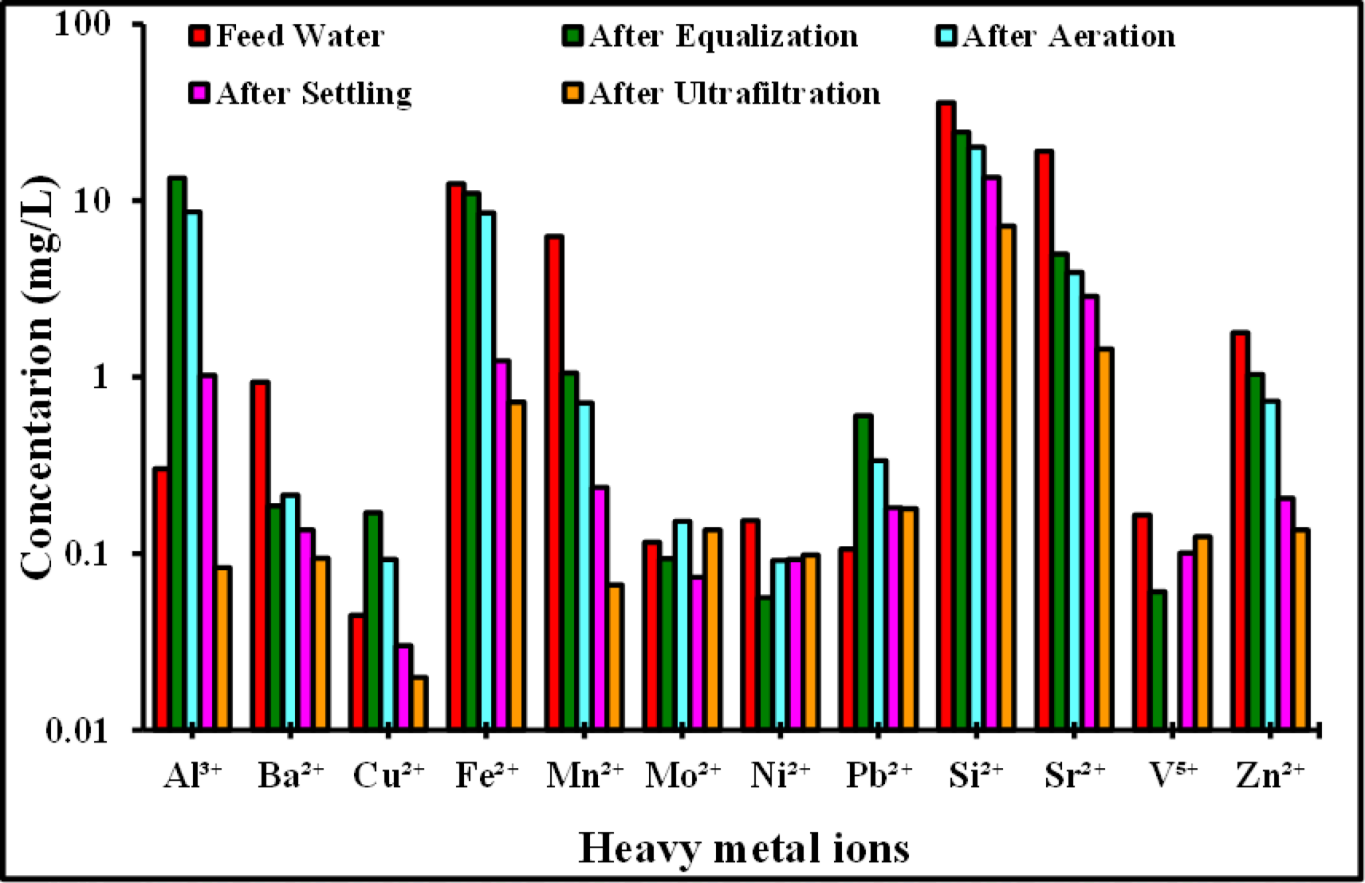
Concentration of heavy metal ions for the feed wastewater and after each stage (Equalisation, Aeration-Moving bed biofilm reactor, Settling, and Ultrafiltration) using municipal wastewater from the Holding Company for Potable Water and Wastewater in Cairo, Egypt. Y-axis shown in logarithmic scale.

Figure 10b shows the removal efficiency after each stage relative to the feed water, in which the water quality steadily improved over the course of the treatment process. Only modest reductions (TDS 8.9%) were noted following equalisation, primarily as a result of stabilisation and partial settling. Overall, the main function of this step was to homogenise the supply water and provide limited removal efficiency. Aeration improved removal, especially for Ca^2+^ (23.8%) and HCO_3_^−^ (37.9%), which were attributed to pH increase and carbonate precipitation from CO_2_ stripping, as shown in Fig. 11b. These findings demonstrate that aeration can lead to lowering carbonate hardness and ionic load to some extent. Settling also decreased precipitated and suspended materials, with removal efficiency for TDS reaching around 24%. Overall clarity and stability were improved by settling, which facilitated the removal of precipitates that formed during aeration. With significant ion removals reaching approximately 45–49% and TDS reductions of 50.7%, ultrafiltration showed the biggest improvement, as shown in Fig. 11b. These findings demonstrate that ultrafiltration is the best step for salt removal and enhancing the quality of water.

Salinity, hardness, and ionic load were all gradually decreased by the treatment train. Equalisation was the main factor in stabilisation; settling eliminated suspended and precipitated particles; aeration caused carbonate precipitation; and ultrafiltration yielded the greatest reduction in dissolved ions and TDS. This methodical removal shows how each phase operates in tandem to provide high-quality treated water.This suggests that ultrafiltration is crucial for polishing the product water and guarantees adherence to water quality regulations.

### Analysis of heavy metal ions

Figure 11 describes the concentration of heavy metal ions for the feed wastewater and after each stage of the compact unit. The feed wastewater sample showed high concentrations of Fe^3+^, Al^3+^, Co^2+^, Cr^3+^, Cd^2+^, Zn^2+^, Li^2+^, Ni^2+^, Cu^2+^, Sr^2+^, Pb^2+^, V^2+^, Mn^2+^, and Si^2+^, possibly indicating minor traces of industrial discharge from the industrial zones located near Cairo, Egypt. However, following the treatment process, it was shown that the compact unit achieved 25–98.9% removal of these metal ions, making it suitable for crop irrigation. The ultrafiltration stage achieved a significant 50.7% decrease in salinity, which was followed by a substantial decrease in the concentrations of heavy element ions, calcium, magnesium, sodium, and sulphate.

The performance of the treatment train varied depending on the metal. Due to coagulant addition or leaching, equalisation resulted in a substantial increase in Al^3+^ (0.30 to 13.47 mg/L) and slight increases in Pb^2+^ and Cu^2+^, while Ba^2+^, Mn^2+^, Sr^2⁺^, and V^5+^ declined. After aeration, Mo^2+^ and Ni^2+^ increased, indicating desorption or chemical change. In addition, aeration facilitated oxidation and partial removal of Fe^2^⁺ (11.01 to 8.51 mg/L) and Mn^2^⁺ (1.06 to 0.71 mg/L), with parallel reductions in Al^3^⁺. By precipitation and solid–liquid separation, settlement further decreased Fe^2+^, Mn^2+^, Sr^2+^, and Si^2+^; nevertheless, some metals (Ni^2+^, V^5+^) increased somewhat as a result of re-dissolution. Ultrafiltration achieved the highest overall removal efficiency, which reduced the amounts of Al^3+^, Fe^2+^, Mn^2+^, Si^2+^, Sr^2+^, and Zn^2+^ to very low levels. In contrast, Mo^2+^, Ni^2+^, Pb^2+^, and V^5+^ were less affected, suggesting that the soluble forms persisted. Overall, the system effectively eliminated metals that were particulate-bound and redox-active, although it was less successful in eliminating soluble transition metals and oxyanions. The ultrafiltration stage demonstrated a strong ability to remove particulate-bound and redox-active species, as confirmed by the high removal efficiencies observed for most metals from the compact unit’s inlet to the final product tank, especially Mn2 + (98.9%), Fe2 + (94.2%), Zn2 + (92.4%), Sr2 + (92.4%), Si2 + (80.1%), and Al3 + (72.5%). V5 + only displayed a minimal reduction (25%), but Cu2 + (55.6%) and Ni2 + (36.1%) demonstrated moderate elimination. The Ba^2+^ (89.9%) showed good removal. Overall, the majority of target metals were successfully polished by ultrafiltration. The results of the water analysis indicate that the treated water met the WHO^44^, FAO^45^, and Egyptian irrigation water quality standards^46^. A comparison between the WHO, FAO, and Egyptian irrigation water standards is included in Table S.3 of the Supplementary Information Files.

### Analysis of wastewater pollutants

**Fig. 12:**
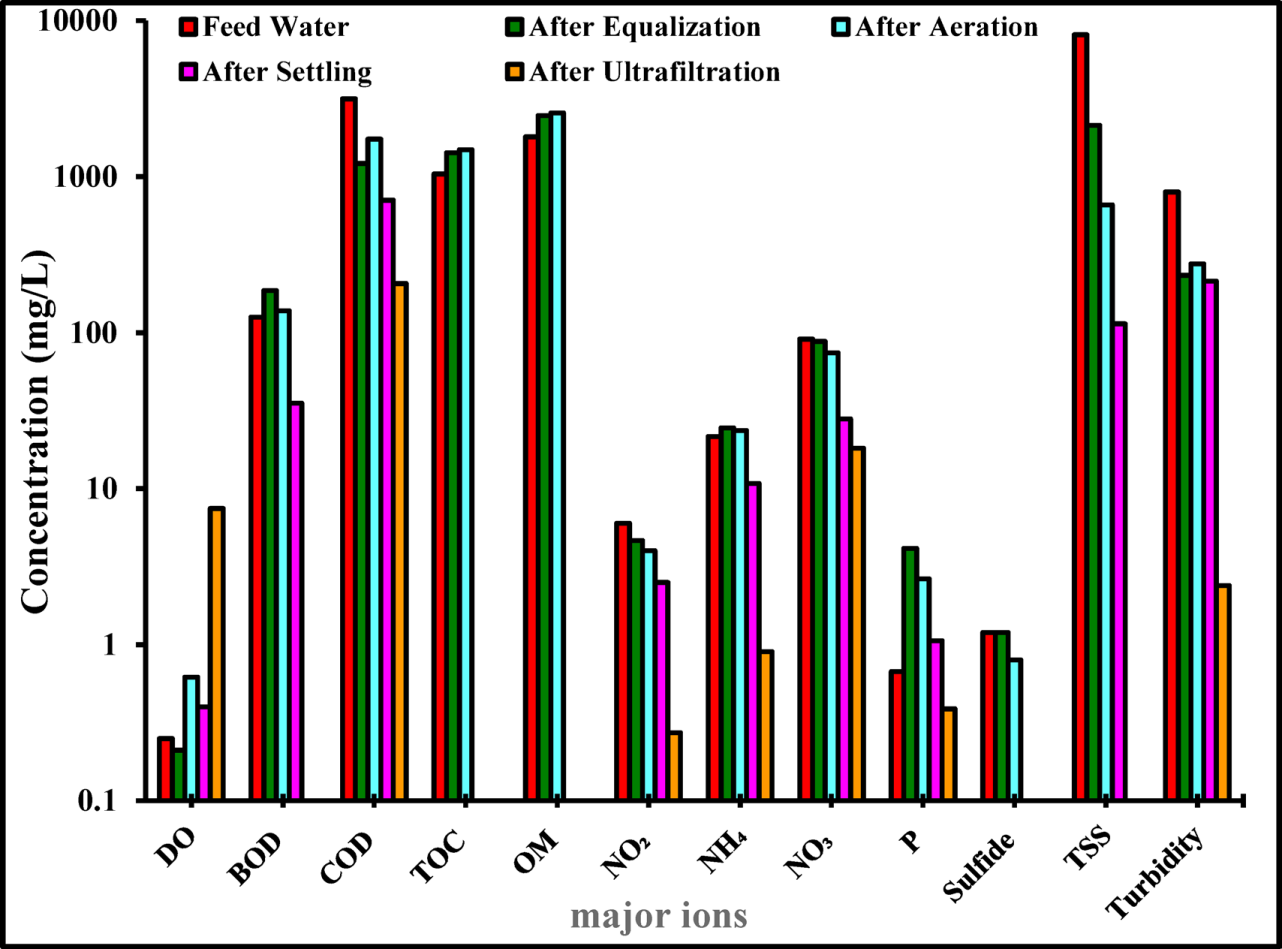
Concentration TSS, turbidity and different parameters for the feed wastewater and after each stage (equalisation, aeration-Moving bed biofilm reactor, settling, and ultrafiltration) using municipal wastewater from the holding company for potable water and wastewater in Cairo, Egypt. Y-axis shown in logarithmic scale.

Figure 12 shows the carbon-based pollutants, including BOD, COD, and TOC. The initial BOD value of the wastewater sample was 126 mg/L, further suggesting the presence of industrial discharge; however, after four stages of purification using the compact unit, it dropped to Below Detection Limit (BDL). Similarly, the TOC concentration in the first water sample was 1043.07 mg/L, and after the purification procedure, it also dropped to BDL, as shown in Fig. 12. As for the COD value, it was 3164 mg/L for the wastewater samples, however, it decreased to around 207 mg/L after the four stages, as shown in Fig. 12. Moreover, the N-containing compounds N–NO₂, N–NH₄, and N–NO_3_, whose concentrations in the initial wastewater sample were 5.98, 21.56, and 91 mg/L, exhibited a considerable reduction to 0.27, 0.9, and 18.2 mg/L, respectively. The concentrations of phosphorus, sulphide, TSS, and turbidity also decreased following treatment with the compact unit, as shown in Fig. 12. It should be noted that the COD concentration of the influent wastewater (3164 mg/L) was much higher than the corresponding BOD₅ value (126 mg/L). This indicates that the wastewater contained a significant fraction of refractory and particulate organic matter, which contributes to COD but is not readily biodegradable within the five-day BOD test. The COD/TOC ratio (~ 3) further confirms the high total organic content and the predominance of non-biodegradable organics in the influent.

### Biological analysis

Table 1 details the results of the biological analysis for the various water samples collected throughout the treatment procedure. The first sample had a total microbial count of 280 CFU/mL and a coliform level of 2400 CFU/100 mL. These counts dropped to 460 CFU/100 mL and 230 CFU/mL, respectively, during the purification process.

**Table 1:** The results of biological analysis for the different water samples collected during the treatment process.

Media used for isolation	Organism	Feed water	After equalisation	After aeration	After settling	After ultrafiltration
MacConky	Pseudomonas	−ve	−ve	−ve	−ve	−ve
XLD	Alcaligenes	−ve	−ve	−ve	−ve	−ve
S.S	Escherichia coli	+ ve	+ ve	+ ve	+ ve	+ ve
	Citrobacter	−ve	−ve	−ve	−ve	−ve
	Salmonella	−ve	−ve	−ve	−ve	−ve
	Shigella	−ve	−ve	−ve	−ve	−ve
	Klebsiella	−ve	−ve	−ve	−ve	−ve
Biochemical test media						
	Proteus	−ve	−ve	−ve	−ve	−ve
TSI						
MIU	Enterobacter	−ve	−ve	−ve	−ve	−ve
MR						
VP						
C (Simmons citrate agar)						
Coliforms count	CFU/100 ml	2400	2400	1100	1100	460
Total microbial count	CFU/ml	280	280	250	250	230

In accordance with APHA Standard Methods for the Examination of Water and Wastewater^47^, the coliform count was determined for microbiological analysis using the Most Probable Number (MPN) method with lauryl tryptose broth, which was validated on Brilliant Green Lactose Bile (BGLB) broth. According to APHA 1998, the total microbial count was ascertained using the standard plate count method on nutrient agar, which was incubated at 35 ± 2 °C for 24 to 48 h. Overall, the results demonstrated that the small unit performed well in treating municipal wastewater. Additionally, the treated water from the final stage meets Grade A requirements, indicating it is suitable for irrigation of edible crops, when the attributes of the treated wastewater are compared to the Egyptian irrigation regulations^48^.

### Discussion on system performance and significance

The proposed compact unit offers multiple benefits. It integrates four WWT technologies into a single compact system to treat and enable the reuse of municipal wastewater. Despite its small footprint, the unit delivers high treatment capacity and provides a cost-effective alternative to centralised WWT systems for agricultural reuse. Its implementation reduces carbon emissions in agricultural communities, improve oxygen levels, and mitigate the negative impacts of climate change. Furthermore, it prevents environmental pollution from wastewater discharge and reduces health risks associated with mixing untreated wastewater with drinking water. The unit also allows for future development for biofuel production from both solid and activated sludge, offering a complete solution that links the water-energy nexus and supports sustainable agriculture.

Table 2 includes a comparison between the performance of the compact unit developed in this study and previous literature. The compact aeration MBBR–MBR system’s performance in this study is in line with and occasionally superior to values documented in the literature. For instance, in hybrid MBBR systems, Javid et al.^49^ found average BOD₅ removals of roughly 88%, but COD removal efficiencies often ranged between 80 and 90%. Moreover, up to 98% BOD₅ and 85–90% COD removal were achieved by a greywater MBBR unit operating on a pilot scale^50^. Similarly, 98.3% BOD₅ and 95.8% COD were removed from ceramic industry effluent treated with an MBBR technique^51^. MBRs have been shown to remove COD and BOD₅ by 92–96%, and at reasonable wastewater loads.

**Table 2:** Results comparison with published literature.

Membrane	Influent conditions	Treatment type	Removal efficiencies	References
Advancement in biological WWT using hybrid MBBR	Municipal/high-strength wastewater	Hybrid MBBR with ~ 500 m^2^/m^3^ media surface area; various HRTs	BOD₅: ~ 88% average removal; COD: somewhat lower but in many cases ~ 80–90% in well-operated setups	^49^
MBBR in greywater treatment efficiency	Greywater with baseline COD ~ 360 mg/L, BOD₅ ~ 127 mg/L	MBBR system	COD: 85–90% removal; BOD₅: ≈ 98% removal; high removal of NH_4_^+^	^50^
Ceramic industry effluent MBBR case study	Highly polluted ceramic industry wastewater	MBBR, with biosurfactants, halophilic/ halotolerant microbes	COD: ~ 95.8% removal; BOD: ~ 98.3% removal; TSS also strongly reduced	^51^
MBR performance	Mixed municipal / industrial influent; OLRs ~ 0.5–1.37 kg COD/m^3^·d	MBR units	COD removal ~ 92–95%; BOD₅ removal ~ 94–96% under steady state; effluent COD < 100 mg/L in many cases, BOD₅ < 30 mg/L in good conditions	^52^
MBBR vs MBR	Across many studies and wastewater types	Meta-analysis of MBBR, MBR, and hybrid systems	For MBBR: BOD removal ~ 87%, COD removal ~ 80% For MBR: BOD removal ~ 88%, COD removal ~ 84% Total nitrogen and total phosphorus nutrients also moderately removed	^53^
MBR from a microbial perspective	Using different MBR-based techniques for the treatment of saline wastewater	MBR-based techniques	Conventional MBR proved to be an efficient technology when the influent salinity was less than 10 g/L NaCl	^54^
Novel compact system for municipal WWT	Integrating an ultrafiltration MBR with mechanical sieving, extended aeration, and a MBBR	Ultrafiltration MBR	Reduction in TDS from 2348.8 mg/L to 1157.2 mg/L and removal of 25–98.9% of the heavy metal ions. BOD and TOC were 126 mg/L and 1043.07 mg/L and dropped to BDL after four stages of purification. COD was 3164 mg/L and decreased to 207 mg/L after the four stages. The initial coliform count and total microbial count were 2400 CFU/100 mL and 280 CFU/mL, respectively, and decreased to 460 CFU/100 mL and 230 CFU/mL, respectively, after treatment	This work

The BOD₅, COD, and TOC were reduced from 126 mg/L to BDL, 3164 mg/L to 207 mg/L (approximately 93–94% elimination), and 1043 mg/L to BDL, respectively, using our compact unit. The resulting effluent quality and removal efficiencies indicate that the compact unit performs at least as effectively as conventional MBBR and MBR systems, confirming its suitability for wastewater reuse applications, even though the influent COD in this study was higher compared to the majority of reported literature.

In terms of fouling and its impact on treatment efficiency, no signs of fouling or performance loss were observed during the experimental operation and testing in this study. This is attributed to the unit’s specific design and operational features. The system operates on a cycle of two hours of treated water production followed by a 15 min backwash for the ultrafiltration membranes to minimise fouling. Additionally, in the event of foaming in the aeration tank, the water level can be adjusted through feed pump control, while a rising suction system continuously removes solids and sludge from the settling tank in semi-liquid form, further reducing fouling. Long-term performance monitoring is recommended in future work to evaluate potential operational challenges associated with each stage of the compact unit. Future research should also include a life cycle and techno-economic assessment to determine the system’s overall environmental sustainability and economic feasibility.

## Conclusions

This study has developed and investigated a novel design for a compact municipal WWT system that is capable of producing water suitable for agriculture. A detailed description has been provided for the system design and testing methodology. Municipal wastewater has been used as feed for the compact unit, and samples have been collected at each stage for chemical analysis at the laboratories of the DRC. The main conclusions are as follows:

- The compact unit effectively reduces major ions from 2348.8 mg/L for the initial wastewater sample TDS to 1157.2 mg/L after treatment through the four stages.
- The compact unit removes 25–98.9% of the heavy metal ions from the feed water. The ultrafiltration stage achieves a significant reduction in salinity, with an overall removal efficiency of around 51%, followed by a corresponding decrease in the concentrations of heavy metal ions, calcium, magnesium, sodium, and sulphate.
- The compact unit improves the sanitary quality of municipal wastewater, in which the initial sample’s coliform count drops by 81% (from 2400 CFU/100 ml to 460 CFU/100 ml) at the end of the process, and the total microbial count decreases by approximately 18% (from 280 CFU/ml to 230 CFU/ml).
- The compact unit reduces BOD₅ and TOC to BDL and achieves a 93–94% reduction in COD (3164 mg/L to 207 mg/L), demonstrating performance comparable to conventional MBBR and MBR systems and confirming its suitability for wastewater reuse, even under high-COD influent conditions.

The compact unit produces Grade A irrigation water suitable for edible crop irrigation and enables biofuel production from solid and activated sludge, providing an added benefit for rural communities. Future work should explore renewable energy integration and efficiency enhancements to develop a fully sustainable water–energy–agriculture solution.

## Supplementary Information

Below is the link to the electronic supplementary material.

Supplementary Material 1

## Data Availability

All data generated or analysed during this study are included in this published article and its Supplementary Information files.

## Abbreviations

APHA: American public health association
BGLB: Brilliant green lactose bile
BDL: Below detection limit
BOD: Biochemical oxygen demand
COD: Chemical oxygen demand
DRC: Desert research centre
EC: Electrical conductivity
EMB: Eosin methylene blue
HDPE: High-density polyethylene
HRT: Hydraulic retention time
MBBR: Moving bed biofilm reactor
MBR: Membrane bioreactor
MPN: Most probable number
NTU: Nephelometric turbidity units
pH: Hydrogen ion concentration
PPM: Parts per million
PVDF: Polyvinylidene fluoride
SRT: Solid retention time
TDS: Total dissolved solids
TSS: Total suspended solids
TOC: Total organic carbon
WWT: Wastewater treatment

## References

[1] Meleshkin, A. V. et al. Phase equilibrium for hydrofluorocarbon R134a hydrate. Hydrate-based desalination of NaCl salt solution. *J. Eng. Thermophys.* **33**(3), 652–662 (2024).

[2] Mito, M.T., et al. Prospects of wind power prediction and variable operation in optimizing wind-powered reverse osmosis operation, In International desalination association. World congress on desalination and water reuse 2019, crossroads to sustainability. 2019, international desalination association (IDA): Dubai, United Arab Emirates.

[3] Basem, A. et al. Technical and financial feasibility of a chemicals recovery and energy and water production from a dairy wastewater treatment plant. *Sci. Rep.* **14**(1), 11143 (2024). 38750120 10.1038/s41598-024-61699-8 PMC11096408

[4] Isawi, H. Using zero nano-valent iron/thin film composite (ZNVI/TFC) membrane for brackish water desalination and purification. *Surfaces Interfaces* **54**, 105237 (2024).

[5] Chahal, C., et al. Chapter two—Pathogen and particle associations in wastewater: Significance and implications for treatment and disinfection processes. In Advances in applied microbiology, (eds S. Sariaslani and G. Michael Gadd), 63–119 (Academic Press, 2016). 10.1016/bs.aambs.2016.08.001 PMC7126130 27926432

[6] El-Aassar, A.-H.M. et al. Design and fabrication of continuous flow photoreactor using semiconductor oxides for degradation of organic pollutants. *J. Water Process Eng.* **32**, 100922 (2019).

[7] WHO. Drinking-water. 2024 [cited 2024 Sep 2024]; Available from: https://www.who.int/news-room/fact-sheets/detail/drinking-water.

[8] Isawi, H., Sadik, M. A. & Nasr, F. A. Combined electrocoagulation/flotation technique and membrane desalination for textile wastewater reuse. *J. Environ. Chem. Eng.* **12**(5), 113661 (2024).

[9] Isawi, H. et al. Semi industrial continuous flow photoreactor for wastewater purification in some polluted areas: Design, manufacture, and socio-economic impacts. *Environ. Nanotechnol. Monit. Manag.* **16**, 100544 (2021).

[10] Mito, M. T. et al. Modular operation of renewable energy-driven reverse osmosis using neural networks for wind speed prediction and scheduling. *Desalination* **567**, 116950 (2023).

[11] Hassan Rashid, M. A. U., Manzoor, M. M. & Mukhtar, S. Urbanization and its effects on water resources: An exploratory analysis. *Asian J. Water Environ. Pollut.* **15**, 67–74 (2018).

[12] Mito, M.T., Optimising the operation of renewable energy-driven reverse osmosis desalination. 2021, Aston University: https://publications.aston.ac.uk/id/eprint/43571/.

[13] El-Khateeb, M. et al. Sustainable municipal wastewater treatment using an innovative integrated compact unit: Microbial communities, parasite removal, and techno-economic analysis. *Annal. Microbiol.* **73**(1), 35 (2023).

[14] Isawi, H. Using Zeolite/Polyvinyl alcohol/sodium alginate nanocomposite beads for removal of some heavy metals from wastewater. *Arab. J. Chem.* **13**(6), 5691–5716 (2020).

[15] Shalaby, T. et al. Geochemistry of El-Salam Canal and the adjacent groundwater in north Sinai, Egypt: An application to a water treatment process using magnetic zeolite nanoparticles. *Appl. Water Sci.* **8**(4), 105 (2018).

[16] Abomostafa, H. M. et al. Advanced photocatalytic degradation of organic pollutants using magnetic nanostructured PVA membrane under solar irradiation. *Surfaces Interfaces* **42**, 103402 (2023).

[17] Sangamnere, R. et al. A critical review of conventional and emerging wastewater treatment technologies. *Sustain. Water Resour. Manag.* **9**(2), 58 (2023).

[18] Isawi, H. Synthesis of graphene oxide-silver (GO-Ag) nanocomposite TFC RO membrane to enhance morphology and separation performances for groundwater desalination, (case study Marsa Alam area- Red sea). *Chem. Eng. Process. Process Intensification* **187**, 109343 (2023).

[19] Isawi, H. Evaluating the performance of different nano-enhanced ultrafiltration membranes for the removal of organic pollutants from wastewater. *J. Water Process Eng.* **31**, 100833 (2019).

[20] Weerasekara, P. C. The united nations world water development report 2017 wastewater: The untapped resource. *Future Food J. Food Agric. Soc.* **5**, 80–81 (2017).

[21] Hellal, M.S., et al. 10—Technologies for wastewater treatment and reuse in Egypt: Prospectives and future challenges. In Handbook of advanced approaches towards pollution prevention and control, (eds Rahman, R.O.A., Hussain, C.M.) 275–310 (Elsevier 2021).

[22] Liang, X. & Yue, X. Challenges facing the management of wastewater treatment systems in Chinese rural areas. *Water Sci. Technol.* **84**(6), 1518–1526 (2021). 34559085 10.2166/wst.2021.332

[23] Angelakis, A. N., Capodaglio, A. G. & Dialynas, E. G. Wastewater management: From ancient greece to modern times and future. *Water* **15**(1), 43 (2023).

[24] Lee, C. et al. Towards decentralized and sustainable water and wastewater treatment systems. *Sci. Rep.* **15**(1), 14331 (2025). 40280991 10.1038/s41598-025-93897-3 PMC12032226

[25] Capodaglio, A. G. Integrated, decentralized wastewater management for resource recovery in rural and Peri-Urban areas. *Resources* **6**(2), 22 (2017).

[26] El-Khateeb, M. A. et al. Integration of UASB and down flow hanging non-woven fabric (DHNW) reactors for the treatment of sewage water, Desalin. *Water Treat.* **164**, 48–55 (2019).

[27] Amer, M. et al. Case study for optimum techno-economic integration of PV and anaerobic digestion for sustainable agri-business. *Energy Rep.* **8**, 362–375 (2022).

[28] Rahman, T. U. et al. The advancement in membrane bioreactor (MBR) technology toward sustainable industrial wastewater management. *Membranes* **13**(2), 181 (2023). 36837685 10.3390/membranes13020181 PMC9965322

[29] Shahot, K.m., et al. Review on biofilm processes for wastewater treatment. 2014.

[30] Metcalf & Eddy, I., et al. Wastewater engineering: Treatment and resource recovery. Fifth ed. 2014: McGraw Hill.

[31] Niazi, S., Separative bioreactor 2011: United States.

[32] Judd, S. The MBR book: Principles and applications of membrane bioreactors for water and wastewater treatment. Second ed. 2010: Elsevier.

[33] Al-Asheh, S., Bagheri, M. & Aidan, A. Membrane bioreactor for wastewater treatment: A review. *Case Stud. Chem. Environ. Eng.* **4**, 100109 (2021).

[34] Hai, F., Membrane biological reactors, (eds. Hai, F.I., Yamamoto, K. and Lee, C.-H.), IWA publishing, UK, 2013 (ISBN: 9781780400655). 2013.

[35] Karczmarczyk, A., Bus, A. & Baryła, A. Assessment of the efficiency, environmental and economic effects of compact type on-site wastewater treatment plants—Results from random testing. *Sustainability* **13**(2), 982 (2021).

[36] Emadzadeh, D. et al. Hybrid forward osmosis/ultrafiltration membrane bag for water purification. *Desalination* **468**, 114071 (2019).

[37] Hem, J.D., Study and interpretation of the chemical characteristics of natural water. In Water Supply Paper. 1985: Reston, VA.

[38] (APHA), A.P.H.A., Standard methods for the examination of water and wastewater. 1998.

[39] (ASTM), A.S.f.T.M. Water and environmental technology. Annual book of ASTM standards. Vol. Vol.11.01 and 11.02 2002, West Conshohocken.

[40] Fishman, M.J. and L.C. Friedman, Methods for determination of inorganic substances in water and fluvial sediments. In Techniques of water-resources investigations, M.J. Fishman, Editor. 1989.

[41] Rainwater, F.H. and L.L. Thatcher, Methods for collection and analysis of water samples. In Water Supply Paper. 1960.

[42] Subramani, T., Elango, L. & Damodarasamy, S. R. Groundwater quality and its suitability for drinking and agricultural use in Chithar River Basin, Tamil Nadu, India. *Environ. Geol.* **47**(8), 1099–1110 (2005).

[43] APHA, Standard Methods for the Examination of Water and Wastewater. 2017, American Public Health Association, American Water Works Association, Water Environment Federation: Washington, D.C.

[44] WHO, Guidelines for drinking water quality. 2017, World Health Organization: Geneva. 28759192

[45] Ayers, R.S. and D.W. Westcot, water quality for agriculture; Food and agriculture organization of the United Nations. 1985.

[46] ECP 501/2015 and Law 48/1982 (Egyptian standards for irrigation and wastewater reuse). 2015, Ministry of Water Resources and Irrigation & EEAA.

[47] APHA, Standard Methods for the Examination of Water and Wastewater., A.P.H. Association, Editor. 2008, American water works association and water environmental federation: Washington DC.

[48] Khaled Abdella Ahmed, A. et al. Comparative study of the Egyptian code for reusing treated wastewater for agriculture. *Sohag Eng. J.* **2**(1), 1–14 (2022).

[49] Javid, A. H. et al. Feasibility of utilizing moving bed biofilm reactor to upgrade and retrofit municipal wastewater treatment plants. *Int. J. Environ. Res.* **7**(4), 963–972 (2013).

[50] Nourredine, H. & Barjenbruch, M. Graywater treatment efficiency and nutrient removal using moving bed biofilm reactor (MBBR) systems: A comprehensive review. *Water* **16**(16), 2330 (2024).

[51] Pethe, A. & Debnath, M. Wastewater treatment using moving bed biofilm reactor technology: A case study of ceramic industry. *Water Environ. Res.* **96**(4), e11026 (2024). 38641883 10.1002/wer.11026

[52] Amin, M. M. et al. Performance evaluation of membrane bioreactor for treating industrial wastewater: A case study in Isfahan Mourchekhurt industrial estate. *Int. J. Environ. Health Eng.* **5**(1), 12 (2016).

[53] Saidulu, D., Majumder, A. & Gupta, A. K. A systematic review of moving bed biofilm reactor, membrane bioreactor, and moving bed membrane bioreactor for wastewater treatment: Comparison of research trends, removal mechanisms, and performance. *J. Environ. Chem. Eng.* **9**, 106112 (2021).

[54] Tan, X. et al. A critical review on saline wastewater treatment by membrane bioreactor (MBR) from a microbial perspective. *Chemosphere* **220**, 1150–1162 (2019). 33395802 10.1016/j.chemosphere.2019.01.027

