# Supplementary material for: Development and testing of a novel compact system for municipal wastewater treatment and irrigation using advanced technologies
Source: Sci Rep. 2025 Dec 8;15:43388. doi: 10.1038/s41598-025-29122-y (PMC12689649; doi:10.1038/s41598-025-29122-y)
Supplement: Supplementary file 1 — Supplementary Material 1 [file 41598_2025_29122_MOESM1_ESM.docx]

**Supplementary material**

**Development and Testing of a Novel Compact System for Municipal Wastewater Treatment and Irrigation using Advanced Technologies**

**Scientific Reports**

Abdel-hameed M. El-Aassar ^a,b^, Heba Isawi ^a,b^, Mohamed E.A. Ali ^a,b^, Hosam A. Shawky ^a,b^, Mohamed T. Mito ^*c^, Selda Oterkus d, Erkan Oterkus ^d^

^a^ Water Treatment and Desalination Unit, Hydrogeochemistry Department, Water Resources and Desert Soils Division, Desert Research Centre, P.O.B. 11753, Cairo, Egypt.

^b^ Egypt Desalination Research Centre of Excellence, Desert Research Centre, Cairo, 11753, Egypt.

^c^ Mechanical Engineering Department, College of Engineering and Technology, Arab Academy for Science, Technology and Maritime Transport, Abu-Qir, Alexandria, Egypt.

^d^ Naval Architecture, Ocean and Marine Engineering, University of Strathclyde, Glasgow, UK.

*Corresponding author: [mohamed.mito@aast.edu](mailto:mohamed.mito@aast.edu)

# Intorduction

This document summarises the experimental data obtained from operating the compact wastewater treatment unit. In addition, it includes a detailed comparison of the WHO, FAO, and Egyptian standards for irrigation water.

# Experimental data and error analysis

Three experiments were conducted to ensure result accuracy and repeatability, and the values presented in the manuscript represent the mean of these trials. Supplementary Table S.1 provides the experimental data and error analysis for major ions at each treatment stage, while supplementary Table S.2 presents the corresponding data and error analysis for heavy metals.

| **Table S.1.** Experimental data and error analsys for major ions at each stage. | | | | | | | | | | | | | | | | | | | | | |
| --- | --- | --- | --- | --- | --- | --- | --- | --- | --- | --- | --- | --- | --- | --- | --- | --- | --- | --- | --- | --- | --- |
| **Parameters** | **Feed water** | | | | | | | | | | | | | | | | | | | | |
|  | **R1** | | | | | **R2** | | | | | **R3** | | **Mean** | | **Final results** | | | **Std.** | | | **Mean ± Std** |
| pH | 7.2 | | | | | 7.15 | | | | | 7.2 | | 7.2 | | 7.2 | | | 0.02887 | | | 7.2 ± 0.02887 |
| EC | 3418 | | | | | 3422 | | | | | 3420 | | 3420 | | 3420 | | | 2.00000 | | | 3420 ± 2.0000 |
| TDS | 2344.0 | | | | | 2348.8 | | | | | 2353.58 | | 2348.8 | | 2348.8 | | | 4.78000 | | | 2348.8 ± 4.78000 |
| Ca ppm | 336.3 | | | | | 335.89 | | | | | 335.81 | | 336 | | 336 | | | 0.26287 | | | 336 ± 0.26287 |
| Mg ppm | 110 | | | | | 108.9 | | | | | 109.15 | | 109.35 | | 109.35 | | | 0.57663 | | | 109.35 ± 0.57663 |
| Na ppm | 241.0 | | | | | 238 | | | | | 241 | | 240 | | 240 | | | 1.73205 | | | 240 ± 1.73205 |
| K ppm | 52 | | | | | 53 | | | | | 54 | | 53 | | 53 | | | 1.00000 | | | 53 ± 1.00000 |
| CO3 ppm | 0 | | | | | 0 | | | | | 0 | | 0 | | Nil | | | 0.00000 | | | 0 ± 0 |
| HCO3 ppm | 576.45 | | | | | 578 | | | | | 584.05 | | 579.5 | | 579.5 | | | 4.01591 | | | 579.5 ± 4.01591 |
| SO4 ppm | 1100.000 | | | | | 1098.3 | | | | | 1101.7 | | 1100 | | 1100 | | | 1.70000 | | | 1100 ± 1.70000 |
| Cl ppm | 216.5 | | | | | 218 | | | | | 227.605 | | 220.7 | | 220.7 | | | 6.02707 | | | 220.7 ± 6.02702 |
| **Parameters** | **After equalisation** | | | | | | | | | | | | | | | | | | | | |
|  | **R1** | | | | **R2** | | | | | **R3** | | | **Mean** | | | **Final results** | **Std** | | **Mean ± Std** | | |
| pH | 7.48 | | | | 7.5 | | | | | 7.52 | | | 7.5 | | | 7.5 | 0.02 | | 7.5 ± 0.02 | | |
| EC | 3178 | | | | 3178 | | | | | 3184 | | | 3180 | | | 3180 | 3.4641016 | | 3180 ± 3.461016 | | |
| TDS | 2123.6 | | | | 2139.2 | | | | | 2154.85 | | | 2139.2 | | | 2139.2 | 15.65 | | 2139.2 ± 15.65 | | |
| Ca ppm | 300 | | | | 303.23 | | | | | 296.77 | | | 300 | | | 300 | 3.23 | | 300 ± 3.23 | | |
| Mg ppm | 100 | | | | 99 | | | | | 99.89 | | | 99.63 | | | 99.63 | 0.5483612 | | 99.63 ± 0.5483612 | | |
| Na ppm | 219.0 | | | | 222 | | | | | 219 | | | 220 | | | 220 | 1.7320508 | | 220 ± 1.7320508 | | |
| K ppm | 54 | | | | 55 | | | | | 53 | | | 54 | | | 54 | 1 | | 54 ± 1.0000 | | |
| CO3 ppm | 0 | | | | 0 | | | | | 0 | | | 0 | | | 0 | 0 | | 0 ± 0 | | |
| HCO3 ppm | 369.05 | | | | 370 | | | | | 377.25 | | | 372.1 | | | 372.1 | 4.4852536 | | 372.1 ± 4.4852536 | | |
| SO4 ppm | 1075.000 | | | | 1080.4 | | | | | 1084.6 | | | 1080 | | | 1080 | 4.8124838 | | 1080 ± 4.8124838 | | |
| Cl ppm | 191.0 | | | | 196.5 | | | | | 210.975 | | | 199.5 | | | 199.5 | 10.307794 | | 199.5 ± 10.307794 | | |
| **Parameters** | **After aeration + moving bed biofilm reactor** | | | | | | | | | | | | | | | | | | | | |
|  | **R1** | **R2** | | | | | **R3** | | | | | | **Mean** | | **Final results** | | | **Std.** | | **Mean ± Std** | |
| pH | 7.6 | 7.65 | | | | | 7.55 | | | | | | 7.6 | | 7.6 | | | 0.05000 | | 7.6 ± 0.0500 | |
| EC | 2791 | 2793 | | | | | 2786 | | | | | | 2790 | | 2790 | | | 3.60555 | | 2790 ± 3.60555 | |
| TDS | 1946.0 | 1948.7 | | | | | 1951.405 | | | | | | 1948.7 | | 1948.7 | | | 2.70500 | | 1948.7 ± 2.70500 | |
| Ca ppm | 260 | 258 | | | | | 250 | | | | | | 256 | | 256 | | | 5.29150 | | 256 ± 5.29150 | |
| Mg ppm | 103.5 | 103.9 | | | | | 106.07 | | | | | | 104.49 | | 104.49 | | | 1.38286 | | 104.49 ± 1.38286 | |
| Na ppm | 180.0 | 182 | | | | | 178 | | | | | | 180 | | 180 | | | 2.00000 | | 180 ± 2.0000 | |
| K ppm | 49 | 51 | | | | | 50 | | | | | | 50 | | 50 | | | 1.00000 | | 50 ± 1.00000 | |
| CO3 ppm | 0 | 0 | | | | | 0 | | | | | | 0 | | 0 | | | 0.00000 | | 0 ± 0 | |
| HCO3 ppm | 358.9 | 360 | | | | | 360.8 | | | | | | 359.9 | | 359.9 | | | 0.95394 | | 359.9 ± 0.95394 | |
| SO4 ppm | 1000.0 | 1000.1 | | | | | 999.9 | | | | | | 1000 | | 1000 | | | 0.10000 | | 1000 ± 0.10000 | |
| Cl ppm | 174.0 | 177.5 | | | | | 183.355 | | | | | | 178.3 | | 178.3 | | | 4.70628 | | 178.3 ± 4.70628 | |
| **Parameters** | **After settling** | | | | | | | | | | | | | | | | | | | | |
|  | **R1** | | | **R2** | | | | | **R3** | | | **Mean** | | **Final results** | | | | **Std.** | **Mean ± Std** | | |
| pH | 7.4 | | | 7.3 | | | | | 7.55 | | | 7.4 | | 7.4 | | | | 0.1258 | 7.4 ± 0.1258 | | |
| EC | 2858 | | | 2861 | | | | | 2861 | | | 2860 | | 2860 | | | | 1.7321 | 2860 ± 1.7321 | | |
| TDS | 1761.5 | | | 1777.2 | | | | | 1792.9 | | | 1777.2 | | 1777.2 | | | | 15.7000 | 1777.2 ± 15.7000 | | |
| Ca ppm | 233 | | | 235 | | | | | 228 | | | 232 | | 232 | | | | 3.6056 | 232 ± 3.6056 | | |
| Mg ppm | 102 | | | 101 | | | | | 103.18 | | | 102.06 | | 102.06 | | | | 1.0912 | 102.06 ±1.0912 | | |
| Na ppm | 150.0 | | | 155 | | | | | 145 | | | 150 | | 150 | | | | 5.0000 | 150 ± 5.0000 | | |
| K ppm | 50 | | | 51 | | | | | 52 | | | 51 | | 51 | | | | 1.0000 | 51 ± 1.0000 | | |
| CO3 ppm | 0 | | | 0 | | | | | 0 | | | 0 | | 0 | | | | 0.0000 | 0 ± 0 | | |
| HCO3 ppm | 345 | | | 343.6 | | | | | 345.35 | | | 344.65 | | 344.65 | | | | 0.9260 | 344.65 ± 0.9260 | | |
| SO4 ppm | 888.000 | | | 895.5 | | | | | 916.5 | | | 900 | | 900 | | | | 14.7733 | 900 ± 14.7733 | | |
| Cl ppm | 166.0 | | | 167.4 | | | | | 176 | | | 169.8 | | 169.8 | | | | 5.4148 | 169.8± 5.4148 | | |
| **Parameters** | **After ultrafiltration** | | | | | | | | | | | | | | | | | | | | |
|  | **R1** | | **R2** | | | | | **R3** | | | | | **Mean** | | **Final results** | | | **Std.** | **Mean ± Std** | | |
| pH | 7.45 | | 7.5 | | | | | 7.55 | | | | | 7.5 | | 7.5 | | | 0.0408 | 7.5 ± 0.0408 | | |
| EC | 1691 | | 1695 | | | | | 1684 | | | | | 1690 | | 1690 | | | 4.5461 | 1690 ± 4.5461 | | |
| TDS | 1145.4 | | 1157.2 | | | | | 1169.025 | | | | | 1157.2 | | 1157.2 | | | 9.6551 | 1157.2 ± 9.6551 | | |
| Ca ppm | 135.76 | | 138 | | | | | 134.24 | | | | | 136 | | 136 | | | 1.5444 | 136 ± 1.5444 | | |
| Mg ppm | 59 | | 59 | | | | | 56.96 | | | | | 58.32 | | 58.32 | | | 0.9617 | 58.32 ± 0.9617 | | |
| Na ppm | 129.0 | | 133 | | | | | 128 | | | | | 130 | | 130 | | | 2.1602 | 130 ± 2.1602 | | |
| K ppm | 27 | | 28 | | | | | 29 | | | | | 28 | | 28 | | | 0.8165 | 28 ± 0.8165 | | |
| CO3 ppm | 0 | | 0 | | | | | 0 | | | | | 0 | | 0 | | | 0.0000 | 0 ± 0 | | |
| HCO3 ppm | 122 | | 123 | | | | | 121 | | | | | 122 | | 122 | | | 0.8165 | 122 ± 0.8165 | | |
| SO4 ppm | 619.000 | | 613.4 | | | | | 642.6 | | | | | 625 | | 625 | | | 12.6533 | 625 ± 12.6533 | | |
| Cl ppm | 114.6 | | 119.3 | | | | | 122.785 | | | | | 118.9 | | 118.9 | | | 3.3474 | 118.9 ± 3.3474 | | |

| **Table S.2.** Experimental data and error analsys for heavy metals at each stage. | | | | | | | | | | | | | | | | | | | | | | | | |
| --- | --- | --- | --- | --- | --- | --- | --- | --- | --- | --- | --- | --- | --- | --- | --- | --- | --- | --- | --- | --- | --- | --- | --- | --- |
| **Element** | **Feed water** | | | | | | | | | | | | | | | | | | | | | | | |
|  | **R1** | | | **R2** | | | | **R3** | | **Mean** | | | | **Final results** | | | **Std.** | | | | | | | **Mean ± Std** |
| Al | 0.303 | | | 0.3018 | | | | 0.3029 | | 0.3026 | | | | 0.3026 | | | 0.00067 | | | | | | | 0.3026 ± 0.0006 |
| Ba | 0.9381 | | | 0.9365 | | | | 0.9383 | | 0.9376 | | | | 0.9376 | | | 0.00099 | | | | | | | 0.9376 ± 0.0010 |
| Cd | -0.0002 | | | 0.0003 | | | | 0.0001 | | 0.0001 | | | | <0.0006 | | | 0.00025 | | | | | | | <0.0006 ± 0.0002 |
| Co | 0.034 | | | 0.0355 | | | | 0.0352 | | 0.0349 | | | | 0.0349 | | | 0.00079 | | | | | | | 0.0349 ± 0.0008 |
| Cr | -0.001 | | | 0.003 | | | | 0.002 | | 0.0013 | | | | <0.01 | | | 0.00208 | | | | | | | <0.01 ± 0.002 |
| Cu | 0.0452 | | | 0.0441 | | | | 0.0451 | | 0.0448 | | | | 0.0448 | | | 0.00061 | | | | | | | 0.0448 ± 0.0006 |
| Fe | 12.46 | | | 12.44 | | | | 12.45 | | 12.4500 | | | | 12.45 | | | 0.01000 | | | | | | | 12.45 ± 0.010 |
| Mn | 6.274 | | | 6.269 | | | | 6.27 | | 6.2710 | | | | 6.271 | | | 0.00265 | | | | | | | 6.271 ± 0.003 |
| Mo | 0.1164 | | | 0.1157 | | | | 0.1159 | | 0.1160 | | | | 0.116 | | | 0.00036 | | | | | | | 0.116 ± 0.0004 |
| Ni | 0.1545 | | | 0.154 | | | | 0.1541 | | 0.1542 | | | | 0.1542 | | | 0.00026 | | | | | | | 0.1542 ± 0.0003 |
| Pb | 0.1063 | | | 0.1066 | | | | 0.1065 | | 0.1065 | | | | 0.1065 | | | 0.00015 | | | | | | | 0.1065 ± 0.0002 |
| Si | 36.02 | | | 36 | | | | 36.01 | | 36.0100 | | | | 36.01 | | | 0.01000 | | | | | | | 36.01 ± 0.010 |
| Sr | 19.08 | | | 19.06 | | | | 19.07 | | 19.0700 | | | | 19.07 | | | 0.01000 | | | | | | | 19.07 ± 0.010 |
| V | 0.1661 | | | 0.1657 | | | | 0.166 | | 0.1659 | | | | 0.1659 | | | 0.00021 | | | | | | | 0.1659 ± 0.0002 |
| Zn | 1.8 | | | 1.787 | | | | 1.78 | | 1.7890 | | | | 1.789 | | | 0.01015 | | | | | | | 1.789 ± 0.01 |
| **Element** | **After equalisation** | | | | | | | | | | | | | | | | | | | | | | | |
|  | **R1** | **R2** | | | | **R3** | | | | | **Mean** | | | | | **Final results** | | **Std** | | | | | **Mean ± Std** | |
| Al | 13.48 | 13.46 | | | | 13.47 | | | | | 13.4700 | | | | | 13.47 | | 0.01 | | | | | 13.47 ± 0.010 | |
| Ba | 0.1869 | 0.1863 | | | | 0.1869 | | | | | 0.1867 | | | | | 0.1867 | | 0.0003464 | | | | | 0.1867 ± 0.0003 | |
| Cd | -0.0002 | 0.0003 | | | | 0.0001 | | | | | 0.0001 | | | | | <0.0006 | | 0.0002517 | | | | | <0.0006 ± 0.0002 | |
| Co | -0.0004 | 0.0002 | | | | -0.0003 | | | | | -0.0002 | | | | | <0.001 | | 0.0003215 | | | | | <0.001 ± 0.0003 | |
| Cr | -0.002 | 0.001 | | | | 0 | | | | | -0.0003 | | | | | <0.01 | | 0.0015275 | | | | | <0.01 ± 0.001 | |
| Cu | 0.1714 | 0.1703 | | | | 0.171 | | | | | 0.1709 | | | | | 0.1709 | | 0.0005568 | | | | | 0.1709 ± 0.0006 | |
| Fe | 11.02 | 11 | | | | 11.01 | | | | | 11.0100 | | | | | 11.01 | | 0.01 | | | | | 11.01 ± 0.010 | |
| Mn | 1.059 | 1.057 | | | | 1.058 | | | | | 1.0580 | | | | | 1.058 | | 0.001 | | | | | 1.058 ± 0.001 | |
| Mo | 0.094 | 0.0932 | | | | 0.0935 | | | | | 0.0936 | | | | | 0.0936 | | 0.0004041 | | | | | 0.0936 ± 0.0004 | |
| Ni | 0.0568 | 0.0562 | | | | 0.0565 | | | | | 0.0565 | | | | | 0.0565 | | 0.0003 | | | | | 0.0565 ± 0.0003 | |
| Pb | 0.6051 | 0.6043 | | | | 0.605 | | | | | 0.6048 | | | | | 0.6048 | | 0.0004359 | | | | | 0.6048 ± 0.0004 | |
| Si | 24.38 | 24.36 | | | | 24.37 | | | | | 24.3700 | | | | | 24.37 | | 0.01 | | | | | 24.37 ± 0.010 | |
| Sr | 4.962 | 4.96 | | | | 4.961 | | | | | 4.9610 | | | | | 4.961 | | 0.001 | | | | | 4.961 ± 0.001 | |
| V | 0.0611 | 0.0606 | | | | 0.061 | | | | | 0.0609 | | | | | 0.0609 | | 0.0002646 | | | | | 0.0609 ± 0.0003 | |
| Zn | 1.037 | 1.035 | | | | 1.036 | | | | | 1.0360 | | | | | 1.036 | | 0.001 | | | | | 1.036 ± 0.001 | |
| **Element** | **After aeration + moving bed biofilm reactor** | | | | | | | | | | | | | | | | | | | | | | | |
|  | **R1** | | **R2** | | | | **R3** | | | | **Mean** | | | | | **Final results** | | **Std.** | | | **Mean ± Std** | | | |
| Al | 8.64 | | 8.63 | | | | 8.65 | | | | 8.640 | | | | | 8.64 | | 0.01000 | | | 8.64 ± 0.010 | | | |
| Ba | 0.2151 | | 0.2145 | | | | 0.2147 | | | | 0.215 | | | | | 0.2148 | | 0.00031 | | | 0.2148 ± 0.0003 | | | |
| Cd | -0.0002 | | 0.0003 | | | | 0.0001 | | | | 0.000 | | | | | <0.0006 | | 0.00025 | | | <0.0006 ± 0.00025 | | | |
| Co | -0.0004 | | 0.0002 | | | | -0.0003 | | | | 0.000 | | | | | <0.001 | | 0.00032 | | | <0.001 ± 0.0003 | | | |
| Cr | -0.002 | | 0.001 | | | | 0 | | | | 0.000 | | | | | <0.01 | | 0.00153 | | | <0.01 ± 0.001 | | | |
| Cu | 0.0931 | | 0.0923 | | | | 0.0927 | | | | 0.093 | | | | | 0.0927 | | 0.00040 | | | 0.0927 ± 0.0004 | | | |
| Fe | 8.52 | | 8.5 | | | | 8.51 | | | | 8.510 | | | | | 8.508 | | 0.01000 | | | 8.508 ± 0.010 | | | |
| Mn | 0.713 | | 0.711 | | | | 0.713 | | | | 0.712 | | | | | 0.7123 | | 0.00115 | | | 0.7123 ± 0.001 | | | |
| Mo | 0.1532 | | 0.1524 | | | | 0.1528 | | | | 0.153 | | | | | 0.1528 | | 0.00040 | | | 0.1528 ± 0.0004 | | | |
| Ni | 0.0923 | | 0.0915 | | | | 0.0919 | | | | 0.092 | | | | | 0.0919 | | 0.00040 | | | 0.0919 ± 0.0004 | | | |
| Pb | 0.3383 | | 0.3375 | | | | 0.3379 | | | | 0.338 | | | | | 0.3379 | | 0.00040 | | | 0.3379 ± 0.0004 | | | |
| Si | 20.15 | | 20.13 | | | | 20.14 | | | | 20.140 | | | | | 20.14 | | 0.01000 | | | 20.14 ± 0.010 | | | |
| Sr | 3.931 | | 3.929 | | | | 3.93 | | | | 3.930 | | | | | 3.93 | | 0.00100 | | | 3.93 ± 0.001 | | | |
| V | -0.002 | | 0.001 | | | | 0 | | | | 0.000 | | | | | <0.01 | | 0.00153 | | | <0.01 ± 0.001 | | | |
| Zn | 0.7322 | | 0.7314 | | | | 0.7318 | | | | 0.732 | | | | | 0.7318 | | 0.00040 | | | 0.7318 ± 0.0004 | | | |
| **Element** | **After settling** | | | | | | | | | | | | | | | | | | | | | | | |
|  | **R1** | | | | **R2** | | | | **R3** | | | | **Mean** | | | **Final results** | | | **Std.** | | | **Mean ± Std** | | |
| Al | 1.023 | | | | 1.021 | | | | 1.022 | | | | 1.022 | | | 1.022 | | | 0.0010 | | | 1.022 ± 0.001 | | |
| Ba | 0.1367 | | | | 0.1363 | | | | 0.1365 | | | | 0.137 | | | 0.1365 | | | 0.0002 | | | 0.1365 ± 0.0002 | | |
| Cd | -0.0002 | | | | 0.0003 | | | | 0.0001 | | | | 0.000 | | | <0.0006 | | | 0.0003 | | | <0.0006 ± 0.0003 | | |
| Co | -0.0004 | | | | 0.0002 | | | | -0.0003 | | | | 0.000 | | | <0.001 | | | 0.0003 | | | <0.001 ± 0.0003 | | |
| Cr | -0.002 | | | | 0.001 | | | | 0 | | | | 0.000 | | | <0.01 | | | 0.0015 | | | <0.01 ± 0.0015 | | |
| Cu | 0.0306 | | | | 0.0298 | | | | 0.0302 | | | | 0.030 | | | 0.0302 | | | 0.0004 | | | 0.0302 ± 0.0004 | | |
| Fe | 1.239 | | | | 1.237 | | | | 1.238 | | | | 1.238 | | | 1.238 | | | 0.0010 | | | 1.238 ± 0.001 | | |
| Mn | 0.238 | | | | 0.236 | | | | 0.238 | | | | 0.237 | | | 0.2373 | | | 0.0012 | | | 0.2373 ± 0.001 | | |
| Mo | 0.074 | | | | 0.0732 | | | | 0.0736 | | | | 0.074 | | | 0.0736 | | | 0.0004 | | | 0.0736 ± 0.0004 | | |
| Ni | 0.0933 | | | | 0.0925 | | | | 0.0929 | | | | 0.093 | | | 0.0929 | | | 0.0004 | | | 0.0929 ± 0.0004 | | |
| Pb | 0.1827 | | | | 0.1819 | | | | 0.1823 | | | | 0.182 | | | 0.1823 | | | 0.0004 | | | 0.1823 ± 0.0004 | | |
| Si | 13.55 | | | | 13.53 | | | | 13.54 | | | | 13.540 | | | 13.54 | | | 0.0100 | | | 13.54 ± 0.010 | | |
| Sr | 2.865 | | | | 2.863 | | | | 2.864 | | | | 2.864 | | | 2.864 | | | 0.0010 | | | 2.864 ± 0.001 | | |
| V | 0.1013 | | | | 0.1007 | | | | 0.101 | | | | 0.101 | | | 0.101 | | | 0.0003 | | | 0.101 ± 0.0003 | | |
| Zn | 0.2065 | | | | 0.2057 | | | | 0.2061 | | | | 0.206 | | | 0.2061 | | | 0.0004 | | | 0.2061 ± 0.0004 | | |
| **Element** | **After ultrafiltration** | | | | | | | | | | | | | | | | | | | | | | | |
|  | **R1** | | | **R2** | | | | **R3** | | | | **Mean** | | | **Final results** | | | | | **Std.** | | | | **Mean ± Std** |
| Al | 0.0835 | | | 0.0831 | | | | 0.0833 | | | | 0.0833 | | | 0.0833 | | | | | 0.0002 | | | | 0.0833 ± 0.0002 |
| Ba | 0.0945 | | | 0.0941 | | | | 0.0943 | | | | 0.0943 | | | 0.0943 | | | | | 0.0002 | | | | 0.0943 ± 0.0002 |
| Cd | -0.0002 | | | 0.0003 | | | | 0.0001 | | | | 0.0001 | | | <0.0006 | | | | | 0.0002 | | | | <0.0006 ± 0.0002 |
| Co | -0.0004 | | | 0.0002 | | | | -0.0003 | | | | -0.0002 | | | <0.001 | | | | | 0.0003 | | | | <0.001 ± 0.0003 |
| Cr | -0.002 | | | 0.001 | | | | 0 | | | | -0.0003 | | | <0.01 | | | | | 0.0012 | | | | <0.01 ± 0.001 |
| Cu | 0.0203 | | | 0.0195 | | | | 0.0199 | | | | 0.0199 | | | 0.0199 | | | | | 0.0003 | | | | 0.0199 ± 0.0003 |
| Fe | 0.724 | | | 0.722 | | | | 0.723 | | | | 0.7230 | | | 0.7231 | | | | | 0.0008 | | | | 0.7231 ± 0.001 |
| Mn | 0.067 | | | 0.066 | | | | 0.067 | | | | 0.0667 | | | 0.0666 | | | | | 0.0005 | | | | 0.0666 ± 0.001 |
| Mo | 0.1364 | | | 0.1356 | | | | 0.136 | | | | 0.1360 | | | 0.136 | | | | | 0.0003 | | | | 0.136 ± 0.0003 |
| Ni | 0.0989 | | | 0.0981 | | | | 0.0985 | | | | 0.0985 | | | 0.0985 | | | | | 0.0003 | | | | 0.0985 ± 0.0003 |
| Pb | 0.1798 | | | 0.179 | | | | 0.1794 | | | | 0.1794 | | | 0.1794 | | | | | 0.0003 | | | | 0.1794 ± 0.0003 |
| Si | 7.182 | | | 7.174 | | | | 7.178 | | | | 7.1780 | | | 7.178 | | | | | 0.0033 | | | | 7.178 ± 0.0033 |
| Sr | 1.443 | | | 1.439 | | | | 1.441 | | | | 1.4410 | | | 1.441 | | | | | 0.0016 | | | | 1.441 ± 0.002 |
| V | 0.1249 | | | 0.1241 | | | | 0.1245 | | | | 0.1245 | | | 0.1245 | | | | | 0.0003 | | | | 0.1245 ± 0.0003 |
| Zn | 0.137 | | | 0.1362 | | | | 0.1366 | | | | 0.1366 | | | 0.1366 | | | | | 0.0003 | | | | 0.1366 ± 0.0003 |

# Standards for irrigation water

Table S.3 inlcudes a detailed comparison of the WHO, FAO, and Egyptian standards for irrigation water [1-3].

| **Table S.3.** The Permissible standered limit for irrigation water using FAO (1970), WHO (2017), and Egyptian (2015) standards for irrigation water quality. | | | | |
| --- | --- | --- | --- | --- |
| **Variable** | **Units** | **FAO (1970) [1]** | **WHO (2017) [2]** | **Egyptian Standard**  **(ECP 501/2015 / Law 48/1982) [3]** |
| **pH** | — | 6 – 8.5 | 6.5 – 9.2 | 6.0 – 8.5 |
| **TDS** | mg/L | 2000 | 1000 | ≤ 2000 |
| **Ca^2+^** |  | 20 | 100 | ≤ 400 |
| **Mg^2+^** |  | 5 | 150 | ≤ 200 |
| **Na^+^** |  | 40 | 200 | ≤ 230 |
| **K^+^** |  | — | 55 | ≤ 50 |
| **HCO₃⁻** |  | 10 | 400 | ≤ 400 |
| **SO_4_^2-^** |  | 20 | 500 | ≤ 500 |
| **Cl^-^** |  | 30 | 250 | ≤ 350 |
| **COD** |  | 90 | 10 | ≤ 100 |
| **Ag^+^** |  | — | 0.1 | ≤ 0.05 |
| **Al^3+^** |  | 5 | 2.9 | ≤ 5 |
| **Cd^2+^** |  | 0.01 | 0.003 | ≤ 0.01 |
| **Co^2+^** |  | 0.05 | 0.11 | ≤ 0.05 |
| **Cr^2+^** |  | 0.1 | 0.05 | ≤ 0.1 |
| **Cu^2+^** |  | 0.2 | 0.05 | ≤ 0.2 |
| **Fe^2+^** |  | 5 | 0.3 | ≤ 5 |
| **Li^2+^** |  | 2.5 | 1.2 | ≤ 2.5 |
| **Mn^2+^** |  | 0.2 | 0.1 | ≤ 0.2 |
| **Ni^2+^** |  | 0.2 | 0.07 | ≤ 0.2 |
| **Pb^2+^** |  | 2 | 0.01 | ≤ 0.1 |
| **Si^2+^** |  | — | 0.03 | ≤ 0.05 |
| **Sr^2+^** |  | — | 7.0 | ≤ 7.0 |
| **V^5+^** |  | 0.1 | 0.5 | ≤ 0.1 |
| **Zn^2+^** |  | 2 | 3 | ≤ 2 |

# References

1. Ayers, R.S. and D.W. Westcot, Water Quality for Agriculture; Food and Agriculture Organization of the United Nations. 1985.

2. WHO, Guidelines for drinking water quality. 2017, World Health Organization: Geneva.

3. ECP 501/2015 and Law 48/1982 (Egyptian standards for irrigation and wastewater reuse). 2015, Ministry of Water Resources and Irrigation & EEAA.
